# An Improved Method for Sampling and Quantitative Protein Analytics of Cerebrospinal Fluid of Individual Mice

**DOI:** 10.1016/j.mcpro.2025.100958

**Published:** 2025-03-27

**Authors:** Athanasios Lourbopoulos, Stephan A. Müller, Georg Jocher, Manfred Wick, Nikolaus Plesnila, Stefan F. Lichtenthaler

**Affiliations:** 1 Laboratory of Experimental Stroke Research, Institute for Stroke and Dementia Research (ISD), LMU University Hospital, Munich, Germany; 2 German Center for Neurodegenerative Diseases (DZNE), Munich, Germany; 3 Neuroproteomics, School of Medicine and Health, Klinikum rechts der Isar, Technical University of Munich, Munich, Germany; 4 Institute of Laboratory Medicine, University Hospital Ludwig Maximilian University, Munich, Germany; 5 Munich Cluster for Systems Neurology (SyNergy), Munich, Germany

**Keywords:** aging, cerebrospinal fluid, CSF collection, method, mouse, neurodegeneration, quantitative proteomics, trauma

## Abstract

The mouse is the species most commonly used in preclinical research, but protein analytics of murine cerebrospinal fluid (CSF) remains challenging because of low sample volumes (often <10 μl) and frequent contaminations with blood. We developed an improved CSF sampling method that allows routine collection of larger volumes (20–30 μl) of pure CSF from individual mice, enabling multiple protein analytical assays from a single sample. Based on cell counts and hemoglobin ELISAs, we provide an easy quality control workflow for obtaining cell- and blood-free murine CSF. Through mass spectrometry-based proteomics using an absolutely quantified external standard, we estimated concentrations for hundreds of mouse CSF proteins. While repeated CSF sampling from the same mouse was possible, it induced CSF proteome changes. Applying the improved method, we found that the mouse CSF proteome remains largely stable over time in wild-type mice, but that amyloid pathology in the 5xFAD mouse model of Alzheimer’s disease massively changes the CSF proteome. Neurofilament light chain and TREM2, markers of neurodegeneration and activated microglia, respectively, were strongly upregulated and validated using immunoassays. In conclusion, our refined murine CSF collection method overcomes previous limitations, allowing multiple quantitative protein analyses for applications in biomedicine.

## Highlights

• Improved mouse cerebrospinal fluid (CSF) collection with up to 30 μl.
• QC to remove blood contaminated CSF samples.
• Repeated CSF sampling from individual mice is possible.
• CSF proteomics of the 5xFAD Alzheimer’s disease mouse model.

## Graphical Abstract

**Figure:**
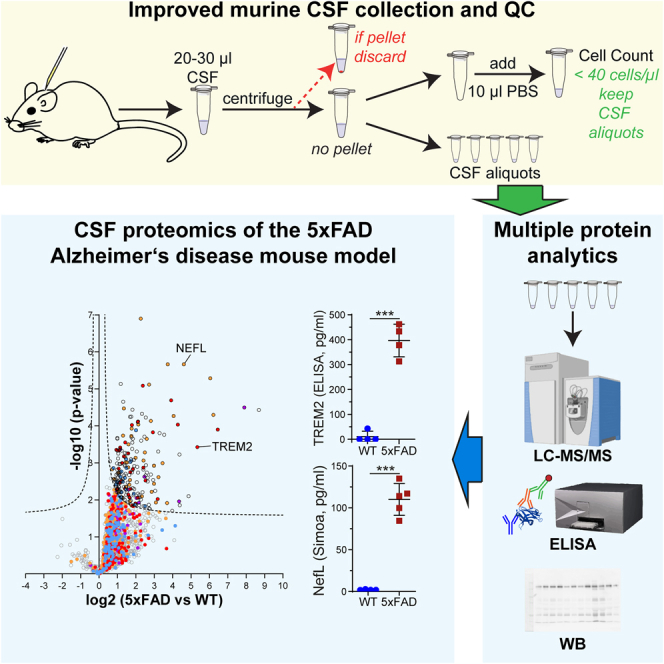

## In Brief

Murine cerebrospinal fluid (CSF) proteomics is challenging due to limited volume and frequent contaminations. This study overcomes previous limitations and presents a method for collecting larger (20–30 μl) volumes of pure CSF from individual mice, enabling multiple protein assays and repeated CSF sampling. A quality control workflow ensures blood- and cell-free CSF. Application to the 5xFAD Alzheimer's model, amyloid pathology significantly altered the CSF proteome, with an increased abundance of neurodegeneration and microglia markers (NEFL and TREM2).

Cerebrospinal fluid (CSF) is the only body fluid in direct contact with the brain and is routinely accessible in a clinical setting. Alterations in CSF composition can inform about physiological and pathophysiological changes occurring in the brain. Consequently, CSF analytics for proteins or cells have become essential for the diagnosis, prognosis, and treatment control of multiple neurological, neurodegenerative, and psychiatric diseases and for a mechanistic understanding of the underlying pathophysiology (1). Examples are the measurement of amyloid β (Aβ), total- and phosphorylated-tau for the diagnosis of AD (2), and neurofilament light chain (NfL) as a marker for neurodegeneration (3). Human CSF is continuously produced at a rate of 300 to 600 μl/min and has a total volume of 90 to 150 ml that is turned over approximately 4 to 6 times per day (4). Human CSF is easily accessible in larger quantities (at least 2–3 ml) in clinical routine, which allows measurement of multiple analytes in parallel from the same collected CSF sample. Human CSF is collected via a needle that directly reaches the subdural space of CSF flow without contact with epidural areas that may potentially contaminate a CSF sample (5).

The mouse is the species most commonly used in preclinical research. CSF protein analytics of single mice is possible, including whole proteome analytics using mass spectrometry (MS) (6), and may enable new biomarker discovery and validation, elucidation of mechanisms of disease pathogenesis, and rapid translation of preclinical results to patients. Despite these promises, there are major challenges to the analysis of mouse cerebrospinal fluid proteins. Compared to humans, mice are smaller (20–40 g body weight), have a lower CSF production (0.32–0.35 μl/min) (7), and have less CSF volume (about 40 μl) which is turned over approximately 12 to 13 times per day (8, 9). Murine CSF collection requires special microsurgical techniques that fall into two approaches. One approach pierces the dura and allows open-CSF-flow on the epidural tissues before collection with a capillary (“epidural” or “open” collection) (10, 11). The other approach comprises a clean puncture of the atlanto-occipital membrane with a capillary and direct collection of the CSF with a capillary reducing the risk of contaminations (“subdural” or “closed” collection) (12). This latter method yields mainly low volumes of CSF (mostly below 10 μl (13, 14)) and the former relatively larger volumes to a maximum of 10 to 20 μl (7, 13, 15) from single mice. Yet, both methods are often below the sample volume requirements of ELISA-based assays. Thus, CSF samples are often pooled from different mice to obtain a sufficient sample volume for protein analytics, which requires larger animal numbers and prevents the generation of single animal-resolved data (16). Moreover, the available murine CSF sampling techniques (especially the “epidural” one) are prone to extra-CSF contamination, such as tissue proteins and blood, which prevents accurate measurements of many proteins in CSF because of the more than 150-fold higher plasma protein concentration (approximately 6.19 ± 0.05 g/dl total protein) compared to CSF (approximately 26 ± 1.5 mg/dl) in mice (17, 18) similar to humans (plasma: 6-7 g/dl; CSF: <45 mg/dl) (19, 20). About 50% of CSF protein quantifications are affected by blood contaminations (21). For human CSF, below 0.01% blood contamination is acceptable for global quantitative proteomics (21, 22). Low blood contaminations may not stain the CSF, making accurate visual recognition impossible and could lead to inaccurate results of CSF protein analytics (22). Currently, no standard procedures are available to monitor blood contaminations of murine CSF.

To overcome limitations in murine CSF analytics, we developed an improved “subdural” (closed), contamination-free sampling method that now allows routine collection of 20 to 30 μl of pure CSF from individual mice and also enables repeated CSF sampling from the same animal. We provide an easy quality control workflow to ensure that mouse CSF is cell- and blood-free. This new method was applied (a) to provide an estimation of absolute concentrations for hundreds of mouse CSF proteins using an absolutely quantified protein standard, (b) to perform repeated sampling of individual mice to reveal resulting changes in the CSF proteome, and (c) to identify CSF proteome changes induced by Aβ plaque formation using the 5xFAD mouse model of Alzheimer’s disease including further protein analytics with ELISA, and Simoa assays.

Collectively, this improved murine CSF collection method overcomes limitations of previous protocols (11, 12, 13, 15), can be controlled to be free of protein or cellular contaminants for mass spectrometry-based biomarker studies, and allows multiple quantitative protein CSF analyses in parallel for many applications in neuroscience.

## Experimental Procedures

### Animal Handling

Adult male C57BL/6 mice (3, 6, and 12 months old, Charles Rivers Laboratories, n = 36 in total) 5xFAD (7 months old, n = 5), and its corresponding wild-type (7 months old, n = 5) male littermate mice were used for the study. The 5xFAD mice model Alzheimer's Disease amyloid pathology; they overexpress mutant human APP695 with the Swedish (K670N, M671L), Florida (I716V), and London (V717I) Familial Alzheimer's Disease (FAD) mutations along with human PS1 harboring two FAD mutations, M146L and L286V (23).

All animals were housed in our animal facility, under a 12/12 h light/dark cycle, and were provided food and water ad libitum. Experiments were conducted according to institutional guidelines of the University of Munich after approval of the Ethical Review Board of the Government of Upper Bavaria (license numbers VET-55.2-1-54-2532-136-2011 and VET-02–20–117).

### Optimized CSF Collection from Cisterna Magna - Surgical Protocol

**Fig. 1:**
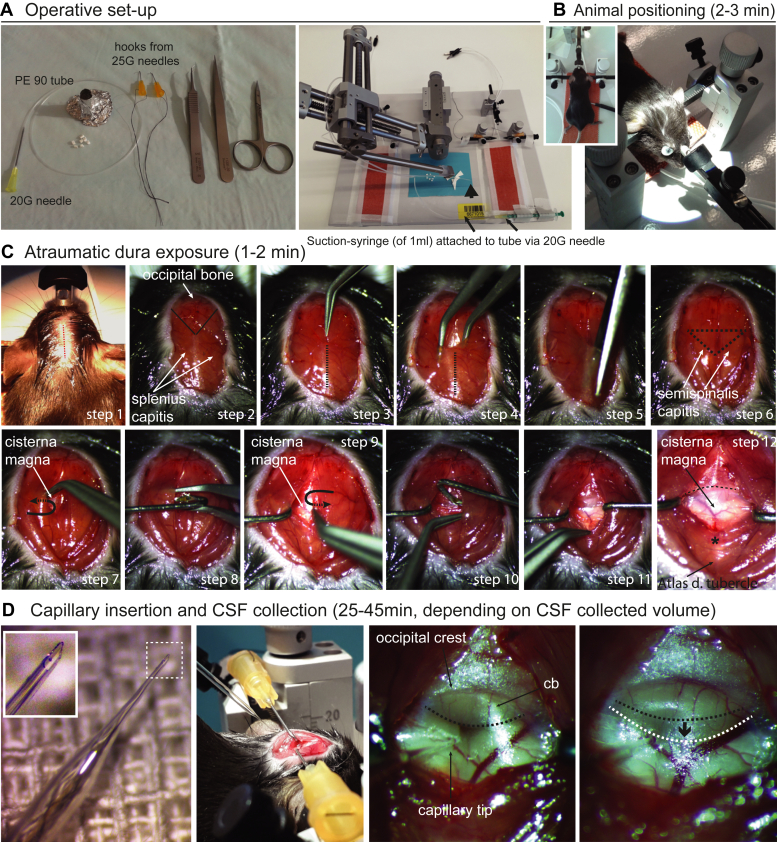
**Stepwise and detailed experimental procedure of CSF collection with the subdural “closed” method.***A*, operative set-up required for cisterna magna puncture and CSF collection (see also text) and stereotactic device with the set-up of suction: 1 ml syringe, 20 G needle, PE90 tubing attached to the 20 G needle (syringe-side, arrows), the glass-capillary for dura puncture (arrowhead) and the pair of self-made orange hooks on ear-bars. *B*, the animal is placed on the ear- and nose-holder (insert in b), and is warmed by a temperature-controlled pad on the stereotaxic floor (the temperature probe is placed under the abdomen of the animal). Note that the head is bent at approximately 135°; whitish Bepanthen eye cream is placed for cornea protection; the head is positioned under a stereotactic microscope. *C*, atraumatic exposure of the Atlanta-occipital membrane (time required: 1–2 min) and detailed single steps (1–12, for description see text). *D*, insertion of the glass capillary (first image and insert magnification: with the optimal shape of the sharp and fine glass-tip) in the cisterna magna (second and third image), followed by slow and stepwise CSF collection (fourth image, see text). Note the angular direction of the capillary towards the cerebropontine depth of the cisterna, which avoids any vessel (second and third images of *D*); collecting of CSF under mild suction results in mild caudal protrusion of the cerebellum (cb, white dashed line and arrow in fourth image of *D*) in comparison to its initial position (*black* dashed line in the third image of *D*).

The collection of the CSF was modified from previously published protocols (13, 15). Initial setup experiments for procedure standardization were performed on 3-month-old C57BL/6 mice (n = 6), which were not used for further analyses. We used the “subdural” method of CSF collection, with no contact with extradural tissues. We describe the protocol in detail below (Fig. 1). The operative set-up and tools needed for the procedure are shown in Figure 1*A* and Supplemental Table S1 (with purchase codes). Mice are anesthetized with ketamine/xylazine (100/10 mg per kg of body weight) and placed on a stereotactic frame with the head bent at approximately 135° (Fig. 1*B*). The surgical procedure is shown in sequential steps (photos) in Figure 1, *C* and *D*. An oil-based cream (such as the Bepanthol Eye-cream) is applied on the skin to pull the cervical fur to both sides of the incision line (Fig. 1*C*, first photo). The skin at the incision line is locally sterilized using 70% alcohol with the aid of a cotton bud. The skin of the dorsal upper cervical region is incised longitudinally for approximately 1.5 cm starting from the base of the skull towards the second cervical vertebra, i.e. starting from a line between the ears downwards for 1.5 cm (red dotted line in Fig. 1*C*, Step 1), thus revealing the occipital bone, the splenius capitis and the respective fascia (Fig. 1*C*, Step 2). Under a dissecting microscope, the superficial cervical muscles (m. splenius) and the thin covering fascia (black lines in Fig. 1*C*, Step 3) are carefully separated by forceps along and up to the median fibrous raphe of the neck (dotted black line in Fig. 1*C*, Steps 3 and 4) (24) without causing tissue bleeding. A sequential midline cut of approximately 0.5–1 cm is done along the median fibrous raphe of the neck to further separate the splenius capitis and hence increase the surgical filed view (Fig. 1*C*, Step 5), taking always care to avoid vessel or muscle cutting which would cause local hemorrhage; here the semispinalis capitis is revealed, under which the atlanto-occipital membrane, i.e. the dura which forms the ceiling of the cisterna magna, is located (Fig. 1*C*, dotted black triangle in Step 6). When necessary, hemostasis is performed by blunt pressure on the muscles, always before exposing the underlying cervical dura.

Next, with the aid of the angled forceps and the self-made hooks (see Fig. 1*A*) the muscles are pulled to the sides to open the surgical field, revealing the atlanto-occipital membrane (see steps 7–11 following in Fig. 1*C*). Initially the angled forceps is inserted just under the semispinalis muscles to guide the insertion of the left hook (Fig. 1*C* step 7, position pointed by a black hooked arrow). By correct positioning of the forceps, the left hook is carefully inserted under the semispinalis capitis (Fig. 1*C* step 8) and its sideward forced retraction reveals the left half part of the atlanto-occipital membrane (Fig. 1*C* step 9). Similarly, the angled forceps guide the insertion of the right hook (Fig. 1*C* steps 9–11). Eventually, by pulling and attaching the strings of the hooks securely to the sides of the earpieces, the atlanto-occipital membrane is non-traumatically revealed (Fig. 1*C* step 12), ready for capillary puncture and CSF collection.

The exposed atlanto-occipital membrane is punctured by a pulled glass capillary. The tip of the capillary has been already cut and beveled by the forceps at an angle of approximately 45° (Fig. 1*D*) under the optical control of the dissecting microscope, just before the glass capillary is connected to the tubing system for suction; the tubing system is a polyethylene PE90 tube connecting the 20-gauge needle (see Fig. 1*A*) of the 1 ml suction syringe to the glass capillary. The latter is carefully stabilized on the head of the stereotactic arm (Fig. 1*A*) just before atlanto-occipital membrane puncturing, to avoid accidental breaking of its fine tip. Once the atlanto-occipital membrane is prepared, the capillary tip is then carefully advanced in position to puncture the atlanto-occipital membrane at an angle of approximately 45^o^ (Fig. 1*D*, second photo): in order to avoid hemorrhages during puncturing, the tip has to point at the depth of the cisterna magna away of the dorsal spinal artery or its branches, as shown in Figure 1*D* (third photo). Once in position, the tip is further advanced to puncture the atlanto-occipital membrane using the micromanipulator controls of the stereotaxic frame. Puncture of the atlanto-occipital membrane by the capillary requires extra force to overcome the resistance of the atlanto-occipital membrane; extra care is necessary to avoid tissue injury by abrupt or excessive tip advancement. Here, some additional head-bending before capillary positioning can stretch the atlanto-occipital membrane and thus ease its puncture by the tip.

Once the tip is successfully inside the cisterna magna (Fig. 1*D*), CSF flows initially freely in the capillary. At that point, the capillary is slightly pulled back for approximately 0.4-0.6 mm -under visual microscopic control gently pull the atlanto-occipital membrane up and increase the volume of the cisterna magna, facilitating further flow of the CSF into the capillary. Still, if the capillary is pulled too much the CSF will leak extradural and the procedure is considered failed due to contamination of the CSF.

An initial free flow of CSF into the glass capillary reflects the normal opening CSF pressure and fills a column of approximately 12 to 15 mm in the capillary. To increase the collected CSF volume, the application of a mild stepwise vacuum via mild suction by the 1 ml syringe (at discrete steps of 20–30 μl every 4-5 min) is necessary. Each suction step has to be applied (a) when the CSF column does not rise anymore in the glass capillary and it pulsates within it, indicating a pressure equilibrium, and (b) when the cisterna has partially refilled. Then, the suction step slightly overcomes the hydrostatic CSF pressure and allows for additional CSF withdrawal (verified by visible CSF withdrawal in the capillary). A partial collapse of the cisterna magna with caudal retraction of the cerebellum is observed and expected, here the black arrow in Figure 1*D* (fourth photo) points at the retraction of the cerebellum from the initial position -black dotted line-to the new one -white dotted line). It is of extreme importance to perform each suction step patiently, to allow for pressure equilibrium and prevent complete caudal sanction of the cerebellum with resulting central herniation and brainstem damage.

After the operation, the animals can be allowed to wake up or are immediately humanely sacrificed. If waking up, they receive analgesia (carprofen) post-operatively and for 2 days, are monitored in their cage for the first 2h post-CSF collection and then can be returned to the animal facility. In the present study, animals were either sacrificed or kept alive after CSF sampling, depending on the experimental design. No animal died during CSF collection. When kept alive, all animals were visually assessed for general signs of discomfort daily for 5 days (e.g. vocalization, humpback, immobility, dirty fur) and all displayed normal activity in their cages (25).

### Experimental Design

All animals were randomized to experimental groups prior to the surgery and all data was analyzed by an investigator blinded to group allocation. Group size for further MS proteomic analysis was calculated based on previous data. We initially setup and improved the surgical procedure of blood-free CSF collection (see above) in C57BL/6 mice (n = 6), then we applied the method on wild-type C57BL/6 and transgenic animals as described below. All experiments followed the same CSF collection and aliquoting procedure.

We used three experimental setups to showcase the validity of our concept: (a) single CSF collections from separate 3, 6, and 12 old C57BL/6 mice (n = 9, 5, and 5 respectively), to study aging-related CSF changes, CSF purity and quantification possibilities, (b) a first CSF collection at 3 months and a second (repeated) CSF collection from the same mice (n = 6), to show the feasibility of multiple, repeated CSF collection from the same mice, and (c) CSF collection from 7-month-old transgenic (5xFAD) mice and their littermates (n = 5 each), to showcase the feasibility of multiple molecular and spectrometric analyses in the collected CSF, also including post-collection contamination control by sensitive hemoglobin-ELISA. In addition, we used pooled CSF from normal C57BL7/6 mice (3 months old, n = 5) for dynamic light scattering (DLS) analysis, as described below. All data are reported according to the ARRIVE criteria (26).

### Human CSF Collection

Anonymized leftover material of human CSF from 8 subjects was provided by the Institute of Laboratory Medicine, University Hospital Ludwig Maximilian University Munich. The subjects did not have a history of head injury, brain ischemia, infarction, hemorrhage, infection, inflammation, or degenerative neurological disease within 6 months before the collection of CSF. Samples were collected non-traumatically without blood contamination. Further handling and centrifugation of CSF were performed using routine clinical procedures. After centrifugation of CSF at 800*g* it was stored at −80 °C until further processing. No personal identifying information was collected, and the samples were fully anonymized using random numbering of tubes. As the samples were anonymized leftover material, explicit informed consent was not required. The use of human biological material for research purposes was in accordance with ethical guidelines, and no institutional ethics approval was necessary for this study. The human CSF analysis abides by the Declaration of Helsinki principles. The ethics committee of the Ludwig Maximilian University Munich approved the study (Project No.: 25–0208).

### Post-Collection Processing of CSF Samples With Quality Control Analysis

The aforementioned procedure yields mouse CSF without macroscopic blood/cell contamination. An initial centrifugation at 10,000 rpm (2000*g*) for 10 min at 4 °C was routinely performed to discard any possible cells/debris according to most available CSF handling protocols. Although no pellet was evident macroscopically after this centrifugation, the supernatant was always carefully removed and an undisturbed, “pellet” of approximately 2 to 3 μl was left behind (putative cells/debris). The supernatant was then immediately aliquoted and stored at −80 °C until further analyzed, while the pellet was reconstituted with PBS to a final volume of 10 μl, where necessary, for further microscopical analysis and quality control (QC).

The QC analysis of collected CSF was performed with the aid of a Neubauer chamber. The samples used were the CSF pellets, reconstituted with PBS at 10 μl of volume. As such, depending on the initially collected CSF volume (20–30 μl), this represented an approximate 2-3x concentration factor compared to the initial CSF volume. We measured the number of cells (N) in the total 25 small Neubauer squares (equal to 0.1 μl of Neubauer volume) under 40x, using routine methodology. The number of cells per μl of collected CSF was then calculated using the formula: “cells/μl = (N x 10)/concentration factor”.

### Simulation Experiments of Blood Contamination

To understand how blood contamination of the CSF would change its microscopical image under the Neubauer chamber, we simulated this via diluted blood in PBS, at 1:100 and 1:10,000 dilutions, representing 1% and 0.01% contaminations respectively. Specifically, the latter dilution equals a cut-off limit previously accepted as “tolerable” in human CSF studies (22). Here, we measured the number of erythrocytes (red blood cells, RBC) using the routine RBC methodology (in 5 small squares in Neubauer) and calculated the cells/μl using the formula “cells/μl = N × 5 × 10”. All results (CSF and blood) are reported as “cells/μl”. All samples were also analyzed for hemoglobin using an ELISA method (see below). Pure mouse plasma from 5 normal C57BL/6 adult mice was used as a reference control for hemoglobin content.

### Dynamic Light Scattering Analysis of Particle Size in Collected CSF

Microparticle analysis of mouse and human CSF was performed with the aid of Dynamic light scattering (DLS) (27, 28, 29). For the analysis, we used a Zetasizer Nano S particle analyzer (Malvern Instruments Ltd) and the corresponding software (Zetasizer 7.03). Solvent-resistant disposable micro cuvettes (UV-Cuvette micro 70 μl, Brand GmbH, Germany, Cat. No. 759200) were used for experiments with a minimum sample volume of 40 μl. The measurements were made at a fixed position with an automatic attenuator and at a controlled temperature. While human CSF volume was adequate for DLS measurements, for mouse analyses we had to collect CSF from extra mice (C57BL/6, n = 5) and use all volumes solely for this purpose. All CSF samples were measured before and after centrifugation at 800 and 2000*g* and diluted at 1:5 with NaCl 0.9% to reach the required volume of 40 μl per cuvette. For each sample, 3 sets of 10 measurements were automated and averaged to provide one average curve result. Peaks are expressed in nm ± std. A calibration of the system was done with nanoparticles of 125 nm diameter (Supplemental Fig. S2*C*).

### Processing of the Mouse and Human CSF Samples for Liquid Chromatography Mass Spectrometry Analysis (LCMS)

Proteolytic digestion of 5 μl CSF aliquots was performed in 50 mM ammonium bicarbonate with 0.1% sodium deoxycholate. Disulfide bonds were reduced and cysteine residues were alkylated by the addition of 2 μl 10 mM dithiothreitol (Biomol) and subsequently 2 μl 55 mM iodoacetamide (Sigma Aldrich). Proteolytic digestion was performed by consecutive digestion with LysC (0.1 μg; 4 h) and trypsin (0.1 μg; 16 h) at room temperature (Promega).

A volume of 4 μl of 8% formic acid (Sigma Aldrich) and 150 μl of 0.1% formic acid (Sigma Aldrich) was added to acidify the samples. Precipitated deoxycholate was removed by centrifugation at 16,000*g* for 10 min at 4 °C. Peptides were desalted by stop-and-go extraction (STAGE) with C18 tips. Samples were dried by vacuum and dissolved in 20 μl 0.1% formic acid in a sonication bath.

Eight out of 20 μl, correlating with a starting amount of 2 μl CSF, were separated on a nanoLC system (EASY-nLC 1000, Proxeon – part of Thermo Scientific) equipped with a PRSO-V1 column oven (Sonation) using an in-house packed C18 column (30 cm × 75 μm ID, ReproSil-Pur 120 C18-AQ, 1.9 μm, Dr Maisch GmbH, Germany) with a binary gradient of water (A) and acetonitrile (B) containing 0.1% formic acid at 50 °C column temperature and a flow of 250 nl/min (0 min, 2% B; 3:30 min, 5% B; 137:30 min, 25% B; 168:30 min, 35% B; 182:30 min, 60% B; 185 min, 95% B; 200 min, 95% B). The nanoLC was coupled online via a nanospray flex ion source (Proxeon – part of Thermo Scientific) to a Q-Exactive mass spectrometer (Thermo Scientific). Full MS spectra were acquired at a resolution of 70,000 (AGC target: 3 × 10^6^, mass range: 300–1400 m/z). The top 10 peptide ions exceeding an intensity of 5 × 10^4^ were chosen for collision-induced dissociation (Resolution: 17,500, AGC target: 1 × 10^5^, isolation window: 2 m/z). A dynamic exclusion of 120 s was used for peptide fragmentation.

CSF samples from 5xFAD and wild-type control mice were analyzed on an EASY-nLC 1000 coupled to a Q-Exactive HF mass spectrometer (Thermo Scientific) equipped with a PRSO-V1 column oven (Sonation, Germany). Peptide separation was performed on the same columns using peptide separation applying a binary gradient of water and 80% acetonitrile (B) for 120 min at a flow rate of 250 nl/min and a column temperature of 50 °C: 3% B 0 min; 6% B 2 min; 30% B 92 min; 44% B 112 min; 75% B 121 min. Samples were analyzed using data-dependent acquisition (DDA) and data-independent acquisition (DIA) injecting 8 out of 20 μl. For DDA, full MS spectra were acquired at a resolution of 120,000 (AGC target: 3 × 10^6^, mass range: 300–1400 m/z). The top 15 peptide ions exceeding an intensity of 5 × 10^4^ were chosen for collision-induced dissociation (Resolution: 15,000, AGC target: 1 × 10^5^, IT: 100 ms, isolation window: 1.6 m/z). A dynamic exclusion of 120 s was used for peptide fragmentation. For DIA, one MS1 full scan was followed by 20 sequential DIA windows with variable width for peptide fragment ion spectra with an overlap of 1 m/z covering a scan range of 300 to 1400 m/z. Full scans were acquired with 120,000 resolution and an AGC target of 5 × 10^6^. Afterward, 20 DIA windows were scanned with a resolution of 30,000 and an AGC of 3 x 10^6^. The maximum IT for fragment ion spectra was set to auto to achieve optimal cycle times.

### LCMS Data Analysis of CSF Samples

Data from DDA runs were analyzed with Maxquant software (maxquant.org, Max-Planck Institute Munich) (30) version 1.5.5.1 or version 2.1.4.0 for 5xFAD CSF. The MS data were searched against a reviewed canonical fasta database of *Mus musculus* from UniProt (download: March 9th 2017, 16,851 entries) or a database with one protein sequence per gene from UniProt (download: January 12th 2023, 21,949 entries) for 5xFAD CSF. Trypsin was defined as protease. Two missed cleavages were allowed for the database search. The option first search was used to recalibrate the peptide masses within a window of 20 ppm. For the main search, peptide and peptide fragment mass tolerances were set to 4.5 and 20 ppm, respectively. Carbamidomethylation of cysteine was defined as a static modification. Acetylation of the protein N-term as well as oxidation of methionine were set as variable modifications. The false discovery rate for both, peptides and proteins, was adjusted to less than 1% using a target and decoy approach (concatenated forward/reverse database). Only unique peptides were used for quantification. Label-free quantification (LFQ) of proteins required at least two ratio counts of unique peptides. Proteins were considered identified if they were detected by at least 2 unique peptides in at least 3 out of n samples of each group, based on LFQ values. The "intensity-based absolute quantification" or iBAQ values were also calculated as previously described (31). We use LFQ values for relative protein quantification among samples and iBAQ values for estimating absolute quantification values, as defined previously (32, 33).

Data from DIA runs of the 5xFAD experiment were analyzed using the software Spectronaut (version 18.3.230830.50606, Biognosys). The directDIA + workflow was used with default settings, but data normalization was disabled. To increase the depth of the spectral library, DDA runs were added for the spectral library generation. The data was searched against a one protein sequence per gene database from UniProt (download: January 12th 2023, 21,949 entries) and the contaminants fasta of Maxquant. Trypsin/P was defined as protease. Two missed cleavages were allowed. Mass tolerances were set to automatic. Oxidation of methionines and acetylation of protein N-termini were set as variable modifications, whereas carbamidomethylation of cyteines was set as a fixed modification. Precursor, peptide, and protein FDRs were set to 0.01. All MS levels were used applying mean precursor and peptide intensities for the protein quantification.

Additionally, DIA data were analyzed with DIA-NN version 1.8 (34). A library-free search was used with the same fasta database including a database of common contaminants such as trypsin and human keratins. Oxidation methionines and acetylation of protein N-termini were set as variable modifications, whereas carbamidomethylation of cyteines was set as fixed modification. Charge states from two to four with a m/z range of 300 to 1400 were considered. Mass accuracy settings were set to automatic. The match between-runs option was applied. Data normalization was disabled.

We also take into consideration that LFQ values are normalized among samples while iBAQ values are not. Imputation of data was performed with the software Perseus version 1.6.14.0 (35) in the cases of 5xFAD vs wild-type (wt) animal analyses to increase the visibility of the 5xFAD pathology versus the wt animals. Only protein groups with a complete quantification profile in at least one experimental group (5xFAD or wt) were considered and missing LFQ data was imputed after log2 transformation according to a normal distribution with a width of 0.3 and a down-shift of 1.8.

We refer to "qualitative changes" of proteins when we study the presence or absence of a protein in all samples of a group. To report "quantitative changes" we use LFQ values (for relative protein changes between 2 groups) or iBAQ values (for absolute quantification of proteins in samples) only when a protein is detected in both compared groups. Volcano plots are used to illustrate relative protein changes (LFQ values) between the experimental groups. A two-sided non-paired Student’s *t* test was used to evaluate the significance of each protein change between two independent groups. For a repeated sampling of the same animals at 3 and 6 months (3moR and 6moR groups), we used correspondingly a paired sample *t* test. For absolute quantification of selected proteins in different samples (mouse or human) we use the log10 transformed iBAQ (intensity-based absolute quantification) values, data that are below the detection limit are presented in all graphs as "zero values".

### Estimation of CSF Protein Concentrations Using the Proteomics Dynamic Range Standard UPS2 in LCMS

For the estimation of absolute CSF protein concentrations, we used the UPS2 kit proteomics dynamic range standard set, which covers 5 orders of magnitude, and performed a calibration based on the iBAQ intensities.

Briefly, the UPS2 standard was dissolved in 20 μl of 50 mM ammonium bicarbonate and 0.1% sodium deoxycholate. The UPS2 standard was digested with the same protocol as the CSF samples. Finally, an amount of 0.5% and 1% of the digested UPS2 standard, which covers a range from 500 to 0.0025 fmol (or 2.5 amol), was analyzed using the same LC-MS/MS method as above. The known amounts of different proteins were used to generate a calibration curve of absolute protein amounts in fmol based on their iBAQ intensities, using a non-linear fit-analysis of data with the least-squares regression (see statistical analysis section). Data outliers were checked and mathematically excluded where necessary before the generation of the calibration curves using the established ROUT method (36) in GraphPad 9.

### Bioinformatics Analysis

To identify proteins and relative biological functions that are enriched in the CSF samples we used the DAVID 6.8 Bioinformatics Resources of NIAID/NIH (https://david.ncifcrf.gov/summary.jsp) (37). As indicated by the DAVID Database, we used the Functional Annotation Clustering to search for enrichment and consequently for major biological functions and pathways standing out in each of our samples in comparison to the background mus musculus proteome. For this purpose, we checked for enrichment of different functional categories (UP-Keywords; Gene Ontology: GOTERM_CC/BP/MF_DIRECT; Pathways: KEGG and REACTOME) (38) separately for proteins with significantly increased and decreased abundance.

Medium classification stringency, similarity term overlap of 3, similarity threshold of 0.5 for Kappa statistics and Enrichment Threshold (EASE) at 0.05 were applied. We selected and report the significantly different protein clusters based on Benjamini *p*-values of *p* < 0.05 enriched for "increased" and "decreased" proteins. The Benjamini *p*-values are adjusted for multiple comparisons to lower the family-wise false discovery rate and, thus, are more conservative than Fisher Exact *p* values.

5xFAD mouse CSF was analyzed with Ingenuity Pathway Analysis (IPA, Qiagen) version 122103623. The target list was generated with a threshold close to the permutation-based FDR curve using a fold-change of 1.5 and a *p*-value of 0.02 as cut-off criteria. The core analysis was performed with default settings using the nervous system as tissue.

### Enzyme-Linked Immunosorbent Assay of Selected Proteins (Hemoglobin, TREM2, NfL, ApoE, and sAPP) in the Mouse or Human CSF

For detection of murine hemoglobin in mouse CSF (where applicable), mouse plasma, and simulation experiments of blood contamination (see above), all samples were analyzed with a sensitive ELISA-kit (#ab157715, Abcam) according to the manufacturer’s instructions and as previously described (39). Briefly, 1 μl of murine CSF was diluted 1:200 and measured in duplicates (of 0.5 μl each); for plasma and simulation experiments we also used 1 μl of the sample. The computation of standard curves, as well as the analysis of samples with unknown concentrations, was conducted employing a four-parameter logistic fit curve, utilizing the resources available on myassays.com.

For the detection of murine TREM2 in CSF of adult C57/BL6 wildtype or 5xFAD mice, the CSF was analyzed using a previously developed ELISA that was set up on the Meso Scale Discovery (MSD) platform as described previously (40). Briefly, a streptavidin-coated small-spot 96-well plate (MSD, #L45SA-2) was incubated overnight in blocking solution (PBS, 0.05% Tween, 3% BSA) at 4 °C, before the plate was incubated with 25 μl of a biotinylated-anti-TREM2 antibody (#BAF1729, biotechne) at a concentration of 0.125 μg/ml for 90 min. The plate was rotated horizontally on a shaker at 300 rpm. After this, the following incubation steps were carried out at room temperature using a horizontal shaker at 300 rpm. The plate was washed 3x with 250 μl of washing buffer (PBS, 0.05% Tween) per well. Standards were prepared in a two-fold serial dilution using recombinant TREM2-FC (#1729-T2, biotechne) ranging from 400 – 12.5 pg/ml. Prior to incubation, the TREM2 standards were denatured by the addition of a denaturation buffer (final conc.: 20 mM Tris-HCL pH 6.8, 0.4% SDS, 4% Glycerol, 0.2% ß-ME, 5 mM EDTA) and boiling for 5 min at 95 °C. The collected CSF was diluted 1:5 in dilution buffer (PBS, 0.05% Tween, 1% BSA, 2 μl/ml protease inhibitor cocktail freshly added). 50 μl of standards and samples were dispensed in the wells and incubated for 120 min. The plate was cleansed as mentioned above, followed by the addition of 50 μl of a 1 μg/ml rat anti-TREM2 detection antibody (#MABN2321, Sigma Aldrich) into the wells, which was left to incubate for 60 min. The plate underwent another round of the previously detailed washing process, after which a goat-anti-rat Sulfo-tag (#R32AH-1, MSD) secondary antibody was introduced into the wells. This secondary antibody was diluted 1:1000 in blocking buffer, with 25 μl of the mixture added to each well, followed by a 60 min incubation period. Following this, the plate was washed twice with washing buffer and twice with PBS, and then 150 μl of a 1x Read buffer (MSD) was poured into the wells. The plate's contents were then read utilizing the internal MSD platform. To calculate the TREM2 levels in the unknown concentration samples, the MSD platform software was employed, utilizing a four-parameter logistic fit curve regression model.

The concentration of NfL in our 5xFAD mouse CSF samples was measured using the NF-light Advantage Assay Kit (Item 103,186) for the Single Molecule Array (SIMOA) immunoassay SR-X (Quanterix). The analysis was carried out following the manufacturer’s protocol. Briefly, 3.4 μl CSF of adult C57/BL6 wildtype or 5xFAD mice was used to determine the levels of Nfl. The computation of standard curves, as well as the analysis of samples with unknown concentrations, was conducted employing a four-parameter logistic fit curve, utilizing the default settings of the software provided by Quanterix.

ApoE and sAPP were measured in human CSF samples using the Apolipoprotein E Human ELISA Kit (#EHAPOE) and Amyloid Precursor Protein Human ELISA Kit (#KHB0051) kits respectively from Life Technologies, following the manufacturer’s protocol in duplicates using a 1:100 dilution of CSF.

### Statistical Analysis

Statistical analysis and graphs are performed with the GraphPad Prism 9. Venn diagrams were constructed using the online tool InteractiVenn (41). Analyses of selected iBAQ values between groups were performed with appropriate parametric or nonparametric tests and post-hoc tests were adjusted for multiple comparisons. Repeated data for 3moR and 6moR groups were analyzed with paired samples *t* test. The UPS2 fmol-iBAQ curve analysis and construction were performed using the Non-linear Fit analysis of data (for a log-log X-Y line) with the least squares regression with appropriate weighting method, to best fit the curve data. Data in text are reported as mean ± SD, unless noted differently; plotted data in graphs are shown as means ± 95% Confidence Interval (±95%CI, unless noted differently) with superimposition of single values where necessary. Level of significance is set at 0.05 for all statistics.

## Results

### Characterization and Quality Control of the Collected Mouse CSF

We first improved the procedure “subdural” and closed CSF collection in C57BL/6 mice to increase the obtainable volume without CSF contact to the potentially contaminating epidural tissues and to reduce the risk of blood contamination. Higher CSF volumes (10–20 μl) have been reported only for contamination-prone techniques, in which the atlanto-occipital membrane of the cisterna magna was incised and the emerging CSF was collected on the epidural surface (i.e. surgically opened, tissue- and fluid-contaminated space) with a capillary, i.e. openly or “epidural” (10, 11). For the collection procedure, we compiled an operative set-up (Fig. 1*A*) including a stereotactic frame (Fig. 1*A*) to fix the mouse head in an optimal position (Fig. 1*B*) and a dissecting microscope to visualize the atlanto-occipital membrane which forms the dorsal border of the cisterna magna for CSF collection (Fig. 1*C*). Critical points for collection of clean CSF are the fixation of the head, the careful exposure and cleaning of the atlanto-occipital membrane in an atraumatic way without micro-bleedings, the tip of the capillary, and the application of suction for CSF sampling. The head of the mouse has to be bent at approximately 135° and fixed stably. The glass tip has to be broken to create a sharp and short bevel (Fig. 1*D*) to be able to pierce the atlanto-occipital membrane without touching the underlying brainstem. The suction in the capillary has to be increased stepwise when the collected CSF-column in the capillary does not increase anymore and CSF starts pulsating, i.e. approximately every 5 min. Eventually, the whole process lasts approximately 25 to 45 min for each animal. The collected volume depends primarily on the collection time (the longer the time, the larger the volume).

**Fig. 2:**
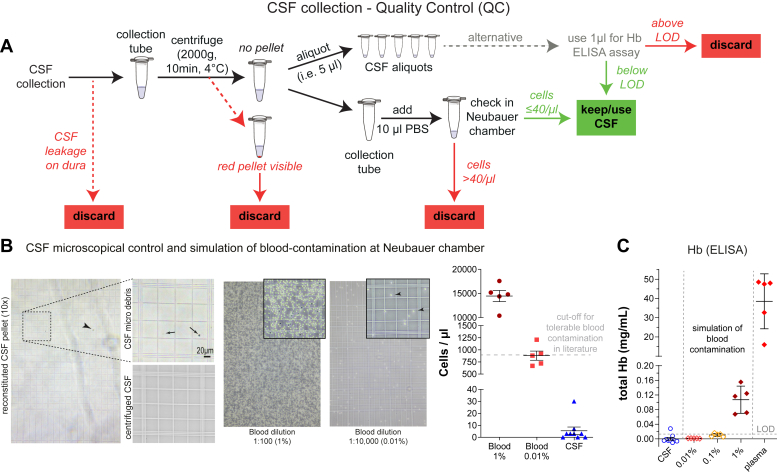
**Quality control for collection and handling of murine CSF samples.***A*, quality control scheme for CSF collection and handling. If a leakage of CSF occurs on the dura, the sample has to be discarded. After the collection, samples are centrifuged at 2000*g* for 10 min at 4 °C. If a *red* pellet is visible, discard the sample. If there is no pellet, aliquot the CSF in low protein binding tubes (i.e. 0.5 ml protein lobind tubes, Eppendorf). Add 10 μl of PBS to the emptied collection tube, which might contain pelleted cells, and check it in a Neubauer chamber. Keep the CSF sample if you have less than 40 cells per μl. Alternatively, i.e. if samples were already collected and stored without performing cell counting, 1 μl of sample can be used for a hemoglobin ELISA (#ab157715, Abcam). Keep the sample if values are below the LOD. *B*, microscopical control and simulation of blood-contamination at Neubauer chamber. The *left* figure shows a reconstituted CSF pellet under a 10x and 20x (magnifications to the *right*) objective. This pellet contains the particles or cells that were in the collected CSF, before centrifugation. Note that apart from few debris (arrows and arrowheads), which is presumably due to dura puncture and is removed by centrifugation, the CSF is largely devoid of cells. Next to it, the simulation of blood contamination with 1% and 0.01% are shown in a Neubauer chamber (under a 10x objective and 20x insert magnification, arrowheads point at erythrocytes). The cell counting results of CSF-cells (in its reconstituted pellet of WT (n = 5) and 5xFAD (n = 5) animals) of 1% blood, 0.01% blood (n = 5, mean ± 95%CI) and the murine CSF (n = 9, mean ± 95%CI) show a 200-fold lower cell content than the 0.01% blood contamination cut-off criteria. *C*, ELISA-based quantification of hemoglobin (Hb) in CSF of WT (n = 5) and 5xFAD (n = 5) animals shows levels below the detection limit of the method for 9/10 samples; for comparison, our simulation experiments of blood contamination show detectable Hb for 1% contamination, and below detection limit for 0.01% (minimal accepted level of CSF contamination with blood (22)) Normal mouse plasma contained 38.5 ± 14.3 mg/L of free Hb.

To assess the purity of the CSF, we developed a protocol based on the results of our study (Fig. 2*A*). After pelleting possibly existing cells in CSF by centrifugation, a visual inspection is applied. A red pellet indicates a strong blood contamination and the sample needs to be discarded. When no red pellet is visible, the sample can be aliquoted while the minimal, potential pellet is kept for microscopic analysis (cell count). Therefore, 10 μl of PBS is added to the collection tube to reconstitute the potential cell pellet and cells are counted with a Neubauer chamber (Fig. 2*B*). We propose this cell count as a gold standard for quality control (QC) of the collected CSF.

To analyze how possible blood contamination, which may happen during CSF collection, would affect the cell count in CSF samples, we carried out simulation experiments diluting mouse blood in PBS. A 1:10,000 blood dilution (0.01%), which is acceptable for proteomics experiments of human CSF (22), resulted in 880 ± 212 cells/μl (mainly RBCs) (Fig. 2*B*). Our collected mouse CSF (of WT and 5xFAD mice) had below 10 cells per μl except one sample with 30 cells. Thus, the collected CSF contained at least 200 times fewer cells, compared to the currently accepted cut-off quality value of 0.01% for cellular contamination for proteomic analyses (21, 22). We conclude that these cells constitute the normal cellular component of murine CSF, similar to human standards, which report 5 cells/μl as normal (5, 42). Given the strong difference in cell counts between a non-contaminated and an artificially blood-contaminated mouse CSF sample, we propose to discard mouse CSF samples with more than 40 cells per μl as being potentially blood-contaminated.

Alternatively, a hemoglobin (Hb) ELISA may be used as quality control, for example for samples that have previously been collected and where the Neubauer chamber analysis is no longer possible (Fig. 2*A*). To validate the utility of a hemoglobin ELISA as an alternative quality control, we used 1 μl of mouse CSF (of WT and 5xFAD mice). Low levels of Hb are expressed endogenously in the brain and may be found in CSF (43), but are expected to increase dramatically upon blood contamination. The Hb ELISA values from 1 μl of CSF (WT and 5xFAD mice) were below the detection limit equal to the “blank values” (Fig. 2*C*). As a control, we simulated how a blood contamination would enhance the CSF concentration of Hb. We artificially diluted mouse blood into PBS at a dilution of 1:100 (1%) and 1:10,000 (0.01%) and compared the ELISA Hb measurement to pure mouse plasma. While the ELISA assay detected Hb in plasma (38.5 ± 14.3 mg/l), at 1% contamination (0.11 ± 0.04 mg/l), and 0.1% (0.011 ± 0.004 mg/l), the Hb values were below the method's detection limit for the artificial 0.01% contamination (21, 22) (Fig. 2*C*). This data indicates that ELISA-based Hb measurement can monitor potential blood contamination of CSF that may affect CSF proteomics.

Additionally, we have analyzed pooled murine and human CSF samples with dynamic light scattering (DLS, Supplemental Fig. S1), because the cell-free CSF obtained after centrifugation may contain microparticles, such as extracellular vesicles. Both, murine and human CSF showed peaks corresponding to microparticles with an approximate size range of 10, 70, and 500 nm, which are much smaller than cells. This is in accordance with data reported in the literature in the range of 26 to 305 nm for human CSF, depending on the measurement method and underlying CSF condition (44).

**Fig. 3:**
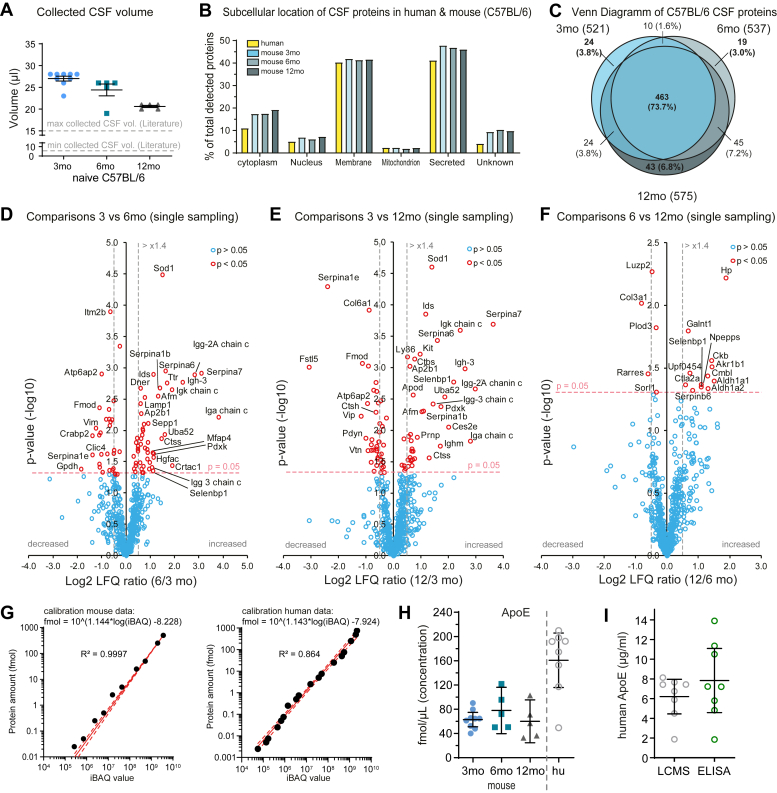
**Quantitative and qualitative characterization of the mouse CSF.***A*, collected CSF sample volumes (μl) for 3 (n = 9), 6 (n = 5) and 12 months (n = 5) old C57BL/6 wild-type mice; *dashed grey lines* show the maximum and minimum reported collected CSF volume using the “subdural” contamination-free method. CSF was collected once from each animal (single-animal samples), no sample pooling was done. *B*, qualitative comparison of mouse CSF to that of human, showing similar subcellular location of proteins in each group. *C*, Venn diagram of detected proteins in mouse CSF of different ages, showing that 72.2% of them are detected through different ages of mice. *D*–*F*, Volcano plots of CSF-proteome comparisons (6 versus 3, 12 versus 3 and 12 versus 6 month-old C57BL/6 mice): x-axes show the Log2 change in protein LFQ relative intensities (increased or decreased abundance) and y-axes the minus log10 *p*-value (two-sided Student’s Ttest) for each single protein of the plot; *red open-circles* indicate significantly changed proteins (*p* < 0.05), blue are not significant. Note that most of the changes occur between 3 and 6 months of age (*D*). *G*, calibrations for iBAQ to fmol for murine and human samples using the UPS2 dynamic range kit as external standard. Digestions of the UPS2 standard were run in the same sample batch for murine and human CSF, respectively. The calibration equations were used to estimate the concentrations of proteins based on their iBAQ intensities. *H*, estimated concentrations (fmol/μl) for Apolipoptotein E (ApoE). *I*, corresponding quantification of ApoE in human CSF via classical ELISA assays show similar results to those of LC-MS. Graphs show the mean ± 95%CI of each group.

Using this method, we obtained 19 to 28 μl (median: 26) of clean CSF from adult wild-type C57BL/6 mice at different ages (3, 6, 12 months) (Fig. 3*A*). This is a significant improvement compared to the previous limit of 10 to 15 μl CSF per mouse reported in the few studies collecting CSF with the “subdural” method (13, 15), while most studies typically collect less than 10 μl of CSF (13, 14).

Collectively, our results indicate that our method surpasses previous (13, 15, 22) quantity and quality standards of CSF murine collection. Quality control for possible blood contamination using the classical Neubauer chamber in the CSF “pellet” is fast, reliable, sensitive, does not use valuable CSF samples, and is translational to human standards (5).

### CSF Proteomics of Aging Mice and Comparison to Human CSF

Next, we applied mass spectrometry-based proteomics to analyze the CSF of 3, 6, 12 months old mice and compared it to human CSF. The majority of CSF proteins were annotated as either secreted soluble proteins or proteolytically released membrane protein ectodomains (Fig. 3*B*), in agreement with relevant previous studies, where less than 15 μl of mouse CSF were obtained using the “subdural” approach (45, 46) (Fig. 3*A*). A similar subcellular distribution of detected proteins was seen in human CSF (Fig. 3*B*) and may be used as another indicator for high-quality CSF with a low number of mitochondrial annotated proteins and a majority of secreted proteins and membrane proteins, which are mainly released by proteolytic ectodomain shedding (47). Whole proteome analysis revealed that the mouse CSF proteome remains largely stable between 3 and 12 months of age, as most proteins had similar abundances at all three time points, as seen in the Venn diagram and the Volcano plots (Fig. 3, *C*–*F*, Supplemental Data S1, Supplemental Fig. S2). Most changes occurred mainly between 3 and 6 months.

The volcano plots showed a normal distribution indicating similar protein amounts (Fig. 3, *D*–*F*), even though we did not use data normalization to be able to reflect potential changes in total protein concentration. Among the changed proteins, the largest changes were detected for different immunoglobulins. Additionally, differences were observed for lysosomal proteins such as cathepsin S (Ctss) and H (Ctsh), iduronate 2-sulfatase (Ids) as well as lysosome-associated membrane glycoprotein 1 (Lamp1). This might be a result of increased lysosomal exocytosis with aging (48). Superoxide dismutase 1 (Sod1), which has an important antioxidant activity, showed the most significant increase comparing 6- and 12- with 3-month-old mice. Mutations in Sod1 are connected with amyotrophic lateral sclerosis (ALS) (49).

In the literature, only a few murine proteins are reported with absolute concentrations values, including total protein (adult, 27.7 ± 0.6 mg/dl, 26 ± 1.5 mg/dl) (17, 18), albumin (17.1 ± 0.7 mg/dl) (17), ApoE (approximately 1.2–6 μg/ml) (50, 51), GFAP (approximately 100–200 ng/l) (16), Apoa1 (approximately 0.60 μg/ml) (51), transthyretin (35 μg/ml in APPswe/PS1A246E mice) (52), and decorin (approximately 10–30 ng/ml) (53), because of the low collection volumes. Therefore, we estimated the absolute concentrations of murine and human CSF proteins using the dynamic range protein standard UPS2 with intensity-based absolute quantification (iBAQ) intensities. The external UPS2 standard was run in the same batch with the murine and human CSF samples to generate experiment-specific calibration curves (Fig. 3*G*). Using these calibration functions, we could estimate the concentrations of 422 murine and 502 human CSF proteins (Supplemental Datas S2 and S3). Exemplary, we show the estimated protein concentration for apolipoprotein E (ApoE), which is linked to cardiovascular and Alzheimer’s disease (54) (Fig. 3*H*). ELISAs for human ApoE yielded comparable protein concentrations to the ones estimated by MS (Fig. 3*I*), and were also in accordance to previous reports for ApoE (approximately 3–11 mg/l for adults (55, 56)). Absolute quantification values are usually provided by mass spectrometry using targeted quantification based on absolutely quantified isotopically labeled peptides. The estimation using the UPS2 standard as a reference—as done in our study—delivers values for hundreds of CSF proteins and might serve as an indicator of protein concentrations for further targeted assay development.

### Repeated Mouse CSF Collection from Single Mice

**Fig. 4:**
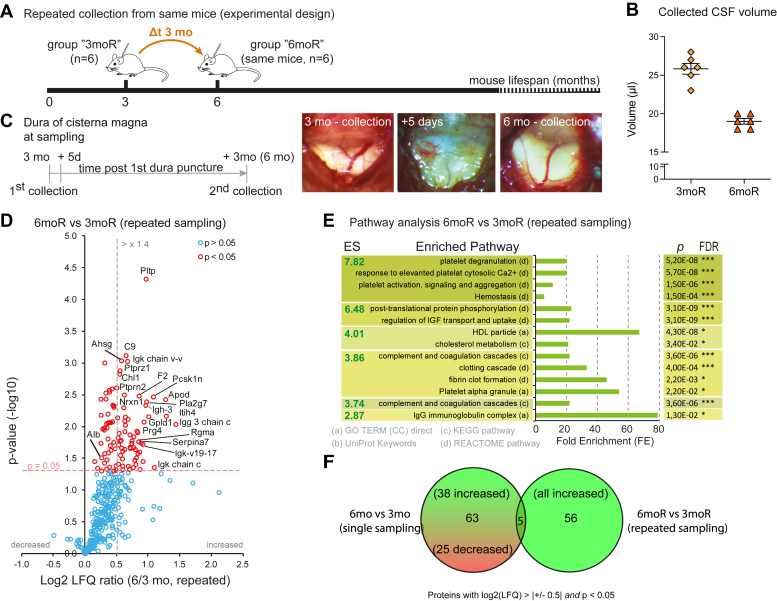
**Repeated mouse CSF collection from single mice.***A*, CSF collection from C57BL/6 mice at 3 months (3moR) followed by a second collection in the same animals at 6 months (repeated collection, 6moR), "repeated collection" experiment (n = 6). *B*, collected CSF volume at each time point (at 3 and then 6 months) from each mouse (mean ± 95%CI). *C*, timeline and representative photos of dural appearance at first CSF collection (3 months, first collection) 5 days after the collection (photo of dura: note the intense fibrotic scarring and the "whitening" of the dura) and at the second collection 3 months after the first (6 months old mice, the dura is now clear and cisterna magna fully reconstituted). *D*, Volcano-plot diagram of CSF proteomic changes between 6 (repeated collection, “6moR”) and 3 (“baseline” collection, “3moR”) months in the same animals (*red open-circles* indicate significantly changed proteins, one sample *t* test with Ho = 0 *p* < 0.05); note an overall increase of proteins mass (positive Log2 LFQ ratios). *E*, functional annotation clustering analysis by DAVID-Database for the repeated sampling cohort only, as this is shown in (*D*) (ES = Enrichment Score); *green boxes* indicate increased pathways (note that the majority of proteins are increased by the repeated collection); the right columns show the corresponding p- (Benajmini correction) and FDR- (false discovery rate) values for each enriched (i.e. changed) terms (∗<0.05, ∗∗<0.01, ∗∗∗<0.001). Despite a fully reconstituted cisterna magna, the CSF showed a significantly changed proteome. *F*, Venn diagram of the significantly altered proteins (proteins with significant log2 LFQ increase of more than ±0.5) in single and repeated sampling cohorts; the two cohorts were significantly different both in terms of changed proteins and the direction of change (increased or decreased).

Furthermore, we tested if repeated CSF sampling of the same mice is possible, and whether this procedure affects the CSF proteome (Fig. 4*A*). We collected CSF from 3-month-old wild-type mice and from the same mice again at 6 months. At 3 months, 25.8 ± 1.7 μl of CSF volume was obtained (Fig. 4*B*), in line with the results from singly punctured mice (Fig. 3*A*). Upon repeated sampling at 6 months, we collected 19.0 ± 0.9 μl of clean CSF. Sampling at intervals shorter than 3 months after the initial sampling did not appear reasonable, because the dura strongly reacted to the initial puncture with fibrosis and thickening and complementary shrinkage of the cisterna magna, which was evident during the first days after the initial puncture (Fig. 4*C*, middle photo) and lasted for at least 4 weeks. After 3 months (at the age of 6 months), the dura and cisterna magna were macroscopically restored (Fig. 4*C*, right photo) and CSF sampling was feasible, in accordance with a previous report (14).

For the proteomic analysis of repeatedly collected CSF samples at 3 and 6 months, we have disabled LFQ normalization to be able to detect potential differences in total protein amount. The CSF proteome analysis (Supplemental Data S1) showed that the repeated sampling at 6 months increased the overall protein content compared to the 3-month time point, which is demonstrated by the right shift of the volcano plot for repeated sampling (Fig. 4*D*) in comparison to the symmetric volcano of single sampling (Fig. 3*D*). The increased protein content is likely a long-term residual response to the fibrosis and thickening of the dura as seen macroscopically (Fig. 4*C*).

Next, we compared the proteome changes detected for repeated and single sampling cohorts, to understand if the repeated sampling at 6 months from the same mice (proteome changes in Fig. 3*D*) changed the CSF differently compared to the single sampling of different mice at 6 months (proteome changes in Fig. 3*D*). Therefore, we analyzed only those proteins with a significantly altered abundance (log2 fold change > |+/− 0.5| and *p* < 0.05). We found that the two experiments showed different proteomic changes (Fig. 4, *E* and *F*): 63 proteins (38 up, 25 down) were uniquely altered for single CSF sampling, while 56 proteins uniquely altered (all increased) for repeated CSF sampling (Fig. 4*F*). On the other hand, they had only a small overlap of five proteins in common (Fig. 4*F*), four of which increased in both samplings (Igh-3, Ig kappa chain C region, Ig gamma-3 chain C region, and Serpina7) and only one changed in the opposite direction (Pcsk1n), being more abundant for repeated CSF sampling and less abundant for single sampling at the 6 versus 3-months time points. Conclusively, CSF sampling of individual mice induces proteome changes, which cannot be explained by aging from three to 6 months alone, but rather by the previous CSF collection at 3 months. This CSF-proteome change should be taken into consideration in CSF-proteome studies when repeated collection is planned.

### CSF Proteomics of the 5xFAD Alzheimer’s Disease Mouse Model

**Fig. 5:**
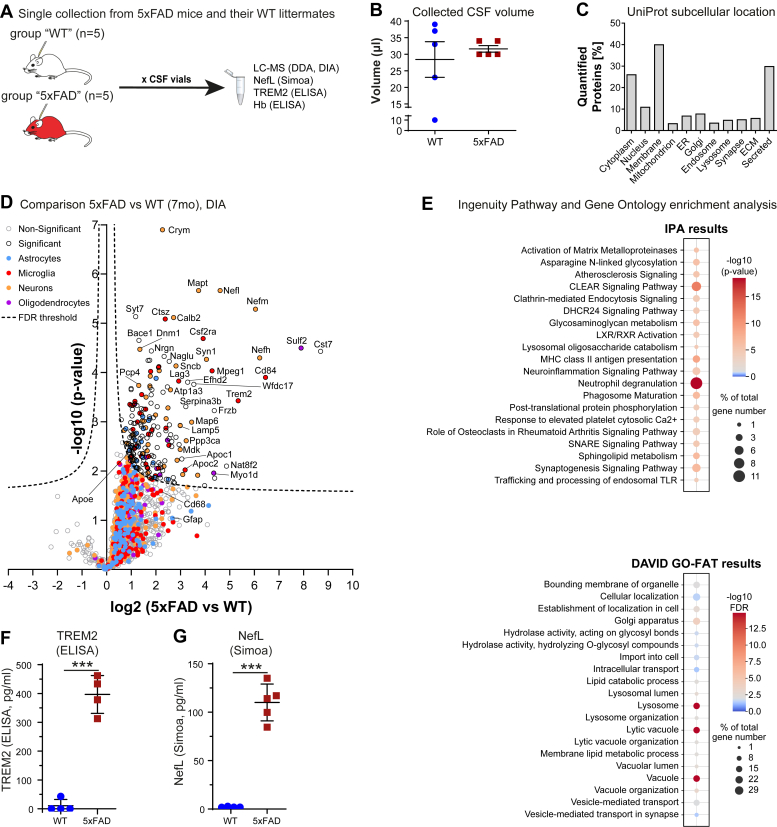
**Multiple protein analytical assays are feasible with single CSF samples.***A*, single-sample CSF collection from WT and 5xFAD animals at 7 months of age (no pooling). CSF was aliquoted in multiple vials (5 μl each) and 4 different assays were used. *B*, collected CSF volume in each group (WT and 5xFAD) from each mouse (mean ± 95%CI of each group; one wt animal fell for technical reasons below the technique’s minimum volume). *C*, UniProt subcellular locations for all relatively quantified proteins. *D*, Volcano-plot of CSF proteomic changes (x-axis: log2 protein LFQ change, y-axis: -log10 *p*-value, two-sided Student’s Ttest) between 5xFAD and wild-type using data-independent analysis. The dashed line indicates a permutation-based FDR correction (*p* = 0.05; s0 = 0.1). Proteins are categorized and labeled by color, based on their major cell type of origin (46, 92). Many neurodegenerative marker proteins show a strong increase, e.g. neurofilaments, Trem2 or MAPT (tau) in 5xFAD animals. *E*, analysis of enriched pathways with Ingenuity Pathway Analysis (IPA) and the GO terms with the functional annotation clustering tool of DAVID-Database. The bubble plots show the percentage of target proteins matching to the pathway or GO terms as dot size and the -log10 transformed *p*-value or FDR as color code, respectively. *F* and *G*, verification of selected quantified proteins, Trem2 (*F*), Nefl (*G*) with MSD ELISA and Simoa, respectively.

Finally, we applied our improved CSF sampling method to showcase that multiple types of protein analyses are possible with one collected sample of mouse CSF. Therefore, we sampled CSF of the 5xFAD (23) transgenic mouse model of Alzheimer’s disease, which expresses human APP and presenilin including five familial AD mutations, and compared its CSF proteome to the one obtained from corresponding wild-type littermates (WT) at 7 months of age followed by validation of key findings using orthogonal methods (ELISA, Simoa) (Fig. 5*A*).

The sampled CSF volume was approximately 30 μl (Fig. 5*B*). For an Hb ELISA, we used 1 μl of the mouse CSF and detected it to be free from blood contamination given that the Hb concentration was below the detection limit and equal to “blank values” (Fig. 2*D*). For a whole proteome analysis, we used 5 μl, of which half of the digested sample was used for data-dependent acquisition (DDA) and data-independent acquisition (DIA) on a Q-Exactive HF mass spectrometer. Due to the pronounced pathology at 7 months in 5xFAD mice, many proteins showed missing quantification values for wild-type control samples, likely because they were below the detection limit, but complete profiles for 5xFAD mice. Therefore, we filtered for proteins with complete quantification data in either the WT or 5xFAD group and imputed missing values with the software Perseus applying a downshift of 1.8 based on a normal distribution (Supplemental Data S4). The DDA data were analyzed with Maxquant, whereas the DIA data were analyzed with Spectronaut, which allows to include DDA data for spectrum matching, and DIA-NN. Spectronaut showed the largest number of average peptide identifications, 1.9-fold higher compared to the DDA analysis, and the best data completeness of 1509 protein groups without missing values, very similar to the DIA-NN analysis. A comparison of the three analyses is shown in Supplemental Fig. S3. Therefore, we decided to perform further data analysis with DIA data analyzed with Spectronaut. Similar to human CSF, the largest group of proteins was annotated as membrane proteins (40%) followed by secreted proteins (30%) and only 3.4% mitochondrial proteins, which might be used as an indicator for cellular contaminations (Fig. 5*C*).

Finally, 1693 protein groups were relatively quantified after data filtering and imputation. The volcano plot indicates increased protein content in the 5xFAD CSF with numerous proteins showing significantly increased abundance (Fig. 5*D*). This strong shift is likely due to increased CSF protein concentration of many proteins in 5xFAD mice. The total protein concentration measured using 2 μl of CSF with the Qubit protein assay, showed a trend of increased protein concentration but was not significant (Supplemental Fig. S4*A*). Albumin, the most abundant protein in CSF also showed a slight, but not significantly increased abundance in 5xFAD CSF (Supplemental Fig. S4*B*), consistent with the total protein concentration measured in the Qubit assay. We conclude that the obvious shift seen in the volcano plot (Fig. 5*D*) for many proteins towards increased abundance in 5xFAD mice might not result from an increase in the total protein concentration but rather from increased concentration of many proteins with a concentration much lower than the abundant CSF proteins, including albumin.

The increase of several microglial proteins such as Trem2, CD84, Lag3, Lyz2, Ctsz, Ctsa, and Ly86 is indicative of a strong microglial activation. The largest fold change of more than 400-fold was detected for Cystatin F (Cst7), a protein found to be enriched for microglia according to the human protein atlas (57). In our previously published microglia proteomics study, Cystatin-F was only sufficiently detected in microglia of the Alzheimer’s mouse models APPPS1 and APP-NLGF-KI showing an increase with ongoing plaque pathology (58). These changes are in line with the fact that 5xFAD mice at 7 months show plaque pathology, microgliosis, cognitive impairment, and synaptic losses (23, 59), and similar to those obtained in another AD mouse line, APPPS1, at 12 months of age (60). Additionally, the neuronal neurofilament proteins L, M, and H (Nefl, Nefm, and Nefh) and tau (MAPT), proposed markers for neurodegeneration showed an increased abundance. Moreover, the inhibitory neuron marker calretinin/calbindin 2 (Calb2) (57) as well as the Purkinje cell protein 4 (PCP4) were more abundant (Fig. 5*D*).

For a bioinformatic analysis of the proteome changes in 5xFAD CSF, we used the software Ingenuity Pathway Analysis to detect potentially altered pathways and disease associations. Filtering for a fold change of at least 1.5 (log2 FC > 0.585) and a *p*-value less than 0.02, a target list of 303 proteins was generated. The top significantly enriched pathways were neutrophil degranulation, which might be involved in AD pathology (61), the CLEAR (Coordinated Lysosomal Expression and Regulation) signaling pathway, MHC class II antigen presentation, phagosome maturation, sphingolipid metabolism, and the synaptogenesis signaling pathway, potentially indicating synaptic loss (Fig 5*E*, Supplemental Fig. S4*K*). Analysis of disease and bio functions showed significant enrichment for several neurologic disorders including Alzheimer’s disease (Supplemental Fig. S4*J*). GO term enrichment analysis with DAVID (Fig. 5*E*) showed a strong significance for lysosome and lytic vacuoles indicating a strong release of lysosomal proteins by exocytosis into the CSF (48, 62). Additionally, protein trafficking and transport as well as lipid metabolic processes and glycosyl bond hydrolase activity showed an enrichment.

Collectively, these changes suggest that damage to neurons and neurodegeneration starts already at 7 months of age, even though neuronal loss was previously described at a later time point (63).

Since our new CSF collection method provided enough CSF volume per mouse for further protein analytics, we validated two protein changes in the same CSF samples by orthogonal methods. We chose Nefl and TREM2, which are under evaluation as potential key markers for neurodegeneration and microglial activation in Alzheimer’s disease (10, 40). Nefl was detected by SIMOA immunoassay using 3.4 μl of single mouse CSF. TREM2 concentration was measured by ELISA in 10 μl of single CSF samples. Similar to the proteomic results (Fig. 5*D*), we found significantly increased levels of Trem2 and Nefl in the CSF of 5xFAD mice (Fig. 5, *F* and *G*), as a result of neurodegeneration and inflammatory microglial activation. The detected increases were in accordance with their corresponding relative quantification by nLC-Mass Spectrometry (Supplemental Fig. S4, *C* and *E*).

Conclusively, this high-quality murine CSF collection protocol provides the opportunity to perform multiple biochemical assays with the same samples. Particularly, mass spectrometry-based proteomics provides a comprehensive overview of CSF protein changes, especially when applying DIA to extend the dynamic range of protein quantification. Our analysis of the 5xFAD CSF proteome demonstrates that murine CSF proteomics offers the possibility to detect neuroinflammatory and -degenerative processes in an unbiased manner.

## Discussion

CSF analytics provides essential information about the status of the healthy and diseased brain. Clinical CSF analysis in humans is widely used to diagnose neurological, psychiatric, and neurodegenerative diseases. Since mice are the most common preclinical animal model, murine CSF analytics have been used to study multiple pathologies in wild-type and transgenic animals (6, 45, 46, 53, 60, 64, 65, 66, 67, 68, 69). The main limitation of such an approach is that CSF analytics are limited by the low volume obtained from single mice and by blood contaminations. These limitations are overcome by our subdural (“closed”) method for sampling murine CSF. The method allows collection of larger volumes (>20 μl) of blood- and cell-free CSF, even upon repeated sampling and enables multiple protein analytical assays in parallel. We also provide an estimation of the absolute concentrations of hundreds of mouse CSF proteins by using the external UPS2 protein quantification standard.

The volume and purity of CSF gained from living mice are critical factors for accurate protein analytics and thus, for robust and reproducible insights into physiological and pathophysiological processes. A volume of 10 to 20 μl is often required for ELISA assays and is important when multiple protein analytical assays are to be run from single samples. A high purity of CSF is essential because contaminations with blood, which has a much higher protein concentration than CSF (22), may profoundly distort CSF protein concentrations. Previously established CSF sampling techniques achieved volumes of up to 20 μl (13, 15), while our improved method allows routine sampling of 20 to 35 μl CSF. Larger volumes of up to 40 μl were only reported in cases, where CSF was collected post-mortem (12) and, consequently, no longer reflected the CSF composition of living mice, or when the CSF was contaminated by inaccurate puncture techniques in which the altlanto-occipital membrane was incised and the emerging CSF was collected with a capillary (extradural or “open” collection) (11, 70).

Our improved method is based on the continuous CSF production in ventricles and its circulation through the CNS (8, 15, 71). We used a stepwise (every 4–6 min) low-grade negative pressure, applied only when the CSF pulsated again in the capillary (i.e. pressure equalization). This subdural (“closed”) collection approach avoided forced CSF suction and possible tissue damage, thus, resulting in a high CSF quality and enabled to collect more than 20 μl CSF within 35 to 45 min in comparison to a passive flow into the capillary, where a volume of approximately 8 μl of CSF fluid was obtained (13, 14).

Our study provides a rough estimation of the concentrations of hundreds of individual CSF proteins (in fmol/μl) based on an external calibration using a commercially available protein reference set covering a wide dynamic range and using intensity-based absolute quantification (iBAQ) (31, 33, 72). A precise concentration determination of individual proteins requires much more expensive, absolutely quantified isotopically labeled spike-in peptides (73) for mass spectrometry or absolutely quantified standards for immunoassays. Therefore, this estimation is particularly helpful when setting up subsequent targeted assays, such as immunoassays that have a limited detection range.

Another outcome of our study is the result from the repeated CSF sampling in mice. We found that the minimal dural trauma due to the puncture for CSF collection induced reactive changes of proteins in the CSF that persisted up to 3 months after the first puncture despite macroscopical restoration of the cisterna magna and dura. The exact reason for the induced proteome changes is not known, but it may be attributed to fibrotic or inflammatory residues of the initial collection, as indicated by DAVID data (Fig. 4) and reported previously (74). Clinically, this may correlate to a widespread meningeal reaction after a single lumbar puncture in some patients, as indicated by reactive gadolinium enhancement of the cranial meninges even weeks after puncture (75). Interestingly, a recent method of CSF collection (76) reports more frequent mouse CSF sampling every 4 days by applying a direct puncture of the cisterna magna using a bent needle without exposing the cisterna magna with an operation at the cost of low sampling volume (5–10 μl). As this method avoids an open surgical “irritation” of the dura it would be interesting to see in future whether and how this minimally invasive approach may affect the CSF proteome during such often-repeated sampling. In any case, the proteomic changes in CSF upon repeated sampling need to be taken into consideration for longitudinal proteomic studies in mice.

Finally, we investigated the CSF proteome of the widely used 5xFAD Alzheimer’s disease mouse model. Using DIA of 2 μl digested CSF per sample injection, we relatively quantified 1693 protein groups including 1509 without missing values, which indicates excellent data completeness. This is within the ID range of 344 to 4012 proteins in other studies (Supplemental Table S5) (7, 46, 53, 60, 64, 65, 76, 77, 78, 79, 80, 81). Noteworthy, analyses with DIA show higher ID numbers than DDA because DIA extends the dynamic range of quantifiable proteins. This is particularly important for CSF analyses with a large dynamic range of protein concentration and albumin as most abundant protein accounting for about 50 to 63% of the CSF total protein amount in mice (17) and 2/3 in humans (82). Deep fractionation of TMT-labeled pooled CSF digests increases the depth of murine CSF proteomics (65), but it requires higher CSF starting volumes and eventually pooling of samples. Additionally, murine CSF proteomics benefits from the recent developments of more sensitive MS instruments such as the timsTOF pro with a larger dynamic detection range (78, 81).

However, the comparison with other mouse proteomics studies is not straightforward based on ID numbers because cell and blood contaminations can strongly increase the IDs. Additionally, neurodegenerative and neuroinflammatory mouse models might lead to increased protein content in the CSF due to secretion and cell degeneration. The most crucial step for high-quality murine CSF proteomics is to avoid contaminations during sample collection. For this purpose, we provide easy quality control using cell counting directly after CSF collection, centrifugation, and aliquoting.

The results of 5xFAD at 7 months of age display the reaction to strong plaque formation, which is accompanied by microgliosis (23). Consequently, compared to wild-type mice, we identified significantly increased abundances of microglia-enriched proteins such as the cathepsins, Csf1r, Csf2ra, Ly86, Lag3, and the microglia activation marker Trem2. These microglial protein signatures are consistent with CSF proteome changes previously identified in another AD mouse model, the APPPS1 model (60), but at a later time point, which are well in line with the faster-progressing pathology in 5xFAD compared to APPPS1 mice (60, 81). The increase of the neurodegeneration markers neurofilaments L, M and H as well as the microtubule-associated protein tau (Mapt) indicate that neurons are already damaged at 7 months in 5xFAD mice (3, 83). Similar changes of Nefm have also been observed in the CSF of 18-month-old APPPS1 and A30PαS mice (60). Increased neurofilament abundance is seen as a sensitive marker for neurodegeneration, even before the massive loss of neurons that characterizes neurodegenerative disease (3, 10). Thus, it is likely that the increased neurofilament levels in the CSF of 5xFAD mice at 7 months indicates that neurodegeneration already starts at 7 months or earlier, whereas overt neuronal loss has been previously detected at 9 months, but was still absent at 6 months of age (59, 63). Moreover, the proposed synapse degeneration marker Snap25 (84, 85) was exclusively quantified in 5xFAD CSF, but it was not significant after data imputation. Increased CSF levels of calbindin 2 (Calb2), also known as calretinin, might be related to the degeneration of calretinin-positive interneurons, which has been described for 12-month-old 5xFAD mice (86). According to single-cell RNA sequencing, calretinin is mainly expressed in inhibitory neurons (57). Hence, CSF calretinin is a novel candidate as a neurodegeneration marker. Moreover, Pcp4 (Purkinje cell protein 4), a known marker for Purkinje cells, was found with an increased abundance possibly indicating degeneration of this cell type. Indeed, loss of cerebellar Purkinje cells have been observed in brains of 5xFAD mice as well as MCI and AD patients (87). Gene ontology and Ingenuity pathway enrichment analyses show a significant alteration of lysosomal proteins pointing towards a release of lysosomal proteins likely by exocytosis (48, 62). The endolysosomal protein PLD3 (2.4-fold increased) is associated with dystrophic neurites, which form around Aβ plaques and display a hall-mark of plaque pathology. In 5xFAD mice, dystrophic neurites have its origin mostly in axon terminals (88). Noteworthy, a recent human CSF proteomics study defining different subtypes of AD, found PLD3 to be highly abundant in subtype 1, which is enriched in proteins related to neuronal hyperplasticity (89). Yet, Crouzin *et al*. (90) showed a complete loss of long-term potentiation in the cortex and therefore deficient cortical neuroplasticity in 6-month-old 5xFAD mice.

In conclusion, our optimized protocol facilitates the collection of large CSF volumes from mice with high quality, which offers the possibility to perform various biochemical analyses using individual CSF samples. This method will help to reduce the number of mice required for CSF studies and will also be instrumental in gaining important new insights into basic and applied neuroscience, such as into brain disorders, the consequences of trauma or drug treatments as well as the function of specific genes/proteins, such as genetic risk factors for brain diseases. The analysis of the AD mouse model 5xFAD impressively demonstrates that murine CSF proteomics has the ability to identify known markers for microglia activation and neurodegeneration but also potentially new candidates.

## Data Availability

The mass spectrometry proteomics data have been deposited to the ProteomeXchange Consortium via the PRIDE (91) partner repository with the dataset identifiers PXD053568 (5xFAD mouse CSF) and PXD053698 (Mouse CSF analysis of 3, 6, and 12 months and human CSF). Annotated peptide fragment spectra for DDA experiments are available at https://msviewer.ucsf.edu/prospector/cgi-bin/msform.cgi?form=msviewer with the search keys **2yiwtza0ye** (Mouse CSF analysis of 3, 6, and 12 months), **tdxfsklhuk** (human CSF) and **ry6drmaqcw** (5xFAD mouse CSF).

## Supporting information

This article contains supporting information. (5, 6, 7, 10, 11, 12, 13, 14, 15, 16, 22, 42, 45, 46, 53, 55, 56, 60, 64, 65, 70, 76, 77, 78, 79, 80, 81, 92, 93, 94, 95, 96, 97, 98, 99, 100, 101, 102, 103, 104, 105, 106)

## Conflict of interest

The authors declare that they have no conflicts of interest with the contents of this article.

## Supporting information

Suppl Data 1R

Suppl Data 2R

Suppl Data 3R

Suppl Data 4R

Supplement_rev
