# Supplementary material for: An Improved Method for Sampling and Quantitative Protein Analytics of Cerebrospinal Fluid of Individual Mice
Source: Mol Cell Proteomics. 2025 Mar 27;24(5):100958. doi: 10.1016/j.mcpro.2025.100958 (PMC12090247; doi:10.1016/j.mcpro.2025.100958)
Supplement: Supplement_rev [file mmc5.pdf]

Supplementary Table 1: Suggested equipment for CSF collection from cisterna magna. Ordering numbers in brackets indicate suggested but not mandatory ordering codes or companies.

Equipment	Description	Supplier	Ordering No.
**Needle 20 Gauge**	needle 20 Gauge for PE tube connection	any available	NA
**Tubing, PE-90/10 (1.27OD x 0.86ID)**	Polyethylene Tubing (3m)	Warner Instruments	64-0754
**1ml syringe**	1ml syringe	any available	NA
**insulin needles (Gauge 25)**	insulin needles (Gauge 25) for self-made hooks (recommended 4 hooks)	any available	NA
**Dumont #5/45 Forceps**	FST Dumont Forceps #5/45 Angled 45o tip 0.1 x 0.06mm	Fine Science Tools	11251-35
**Dumont Forceps Dumoxel**	FST Dumont #3 Forceps	Fine Science Tools	11231-30
**Bonn Scissors**	Straight sharp/sharp tip, 9cm length scissor	Fine Science Tools	14184-09
**Stereotaxic Frame for Mice**	Just for Mouse Stereotaxic Instrument (or other equivalent)	Stoelting (or other equivalent)	(51730)
**Heating pad**	Heating pad for stereotaxic frame	Stoelting (or other equivalent)	(53850M)
**Eppendorf Protein LoBind tubes 0.5ml**	0.5 mL tubes with low protein binding	Sigma-Aldrich (or other equivalent)	(EP0030108094)
**Glass capillaries**	Borosilicate Glass OD 1.0mm, ID 0.75mm, 10cm length	Sutter Instrument	B100-75-10
**Capillary puller**	P-1000 Micropipette Puller (or other equivalent)	(Sutter Instrument)	(P-1000)
**Ketamin-xylazin**	ketamin-xylazin 100:10 mg/kg mixture	any available	NA

Supplementary Table 2: Estimated concentrations of abundant proteins in normal human CSF (n=8), as well as proteins at iBAQ range of 10^7. NA = data not available; "values in literature" indicate (where present) the reported range of mean± SD (or SE according to the reference), for Hemoglobin data "<...>" indicates values that have to be interpreted with caution.

Protein ID	Protein names (human)	Gene names	iBAQ value (± SD)	Estimated values in fmol/µL (± SD)	Estimated values in mg/l (± SD)	Values in literature (mean ± SD or SE)
**P02766**	Transthyretin	TTR	1.57E+09 ± 1.47E+08	193.1 ± 24.5	3.1±0.4 mg/l	15.8 (5.8-26.1) mg/l [1]
**P02649**	Apolipoprotein E	APOE	1.48E+09 ± 4.56E+08	181.4 ± 61.4	6.5±2.2 mg/l	3-9 ± 2-3 mg/l [2, 3]
**P02787**	Serotransferrin	TF	7.23E+08 ± 1.64E+08	79.8 ± 20.6	6.1±1.6 mg/l	NA
**P10909**	Clusterin	CLU	8.45E+08 ± 2.58E+08	95.8 ± 32.4	5.0±1.7 mg/l	1.9 ± 0.036 mg/l [4]
**P01024**	Complement 3	C3	2.33E+08 ± 6.37E+07	21.9 ± 6.8	4.1±1.3 mg/l	1.8 (0.8-3.3) mg/l [5]
**P00441**	Superoxide dismutase [Cu-Zn]	SOD1	7.14E+07 ±3.26E+07	5.73 ±2.99	0.09±0.05 mg/l	0.135 ± 0.047 mg/l [6]
**P05067**	Amyloid beta A4 protein	APP	1.00E+08 ±3.64E+07	8.42 ± 3.42	0.73±0.3 mg/l	0.75 mg/l [7]
**P31946**	14-3-3 protein β/α	YWHAB	2.41E+05 ± 9.06E+04	0.009 ±0.004	0.2±0.1 µg/l	NA for healthy subjects
**P01031**	Complement C5	C5	8.23E+06 ± 5.31E+06	0.49 ± 0.36	0.09±0.07 mg/l	Approx. 0.2±0.1 mg/l [8]
**P02790**	Hemopexin	HPX	9.10E+08± 2.00E+08	103.8±25.9	5.4±1.3 mg/l	22.6 mg/l [9]
**P13521**	Secretogranin-2	SCG2	2.23E+07 ± 9.62E+06	1.51±0.72	0.11±0.05 mg/l	Approx. 1.8 fmol/µl [10]
**Q02818**	Nucleobindin-1	NUCB1	9.07E+06 ± 2.38E+06	0.54±0.16	0.03±0.01 mg/l	NA
**Q99983**	Osteomodulin	OMD	1.23E+07 ± 6.52E+06	0.77±0.47	0.04±0.02 mg/l	NA
**Q06828**	Fibromodulin	FMOD	5.94E+06 ± 2.33E+06	0.33±0.15	0.014±0.006 mg/l	NA
**P68871**	Hemoglobin subunit beta	HBB; HBD	1.00E+07 ±2.27E+07	0.74 ±1.77	0.012±0.028 mg/l	*<3mg/dl>* [11]

Supplementary table 3: Comparisons of main CSF collection methods in mice (open epidural, closed subdural or other), with reported collected volumes, collection frequency, quality control measures (QC), pooling of CSF for analysis or use of single samples, and performed analyses (numbers and types). Human data and studies are presented here as the "gold standard" for comparison. QC NR = Quality control is not reported

Indicative Study	Species	CSF collection method	collected volume (range)	Collection frequency	Normal Cells/µl, QC	Single or pooled CSF sample analysis	Reported CSF analyses
**Clinical routine, lumbar puncture[12, 13] -Gold standard-**	human	"closed", subdural	0.5 to ≥ 40ml	Single/ repeated	0-5/µl	single	large volumes (ml), unlimited number of analyses
**Barkovits K et al 2020[14]**	human	"closed", subdural	Not reported, in range of human (>1ml)	Single/ repeated	0-8/µl, Hb as QC	single	ELISA, Combur10 Test, mass spectrometry
**DeMattos RB et al 2002[15]**	mouse	"open", epidural	15,4 – 17,8µl	Single, terminal	ApoB levels, macroscopic for blood	single	1x WB, 1x RIA
**Maia LF et al 2013[16].**	mouse	"open", epidural	15-20 µl	Single, terminal	Not reported, macroscopic for blood	single	2x ECL-immunoassay, 1x ELISA
**Cunningham R et al, 2013[17]**	mouse	"open", epidural	10µl	Single, terminal	Not reported	pooled	1x ELISA, mass spectrometry
**Bacioglu M et al 2016[18]**	mouse	"open", epidural	15-20 µl	Single, terminal	Not reported, macroscopic for blood	single	1x ECL-immunoassay
**Sakic B 2019[19]**	mouse	Post-mortem subdural	Up to 40µl, post-mortem	Single, terminal	Not reported	single	Not reported
**Smith JS et al 2014[20]**	mouse	Lateral ventricle	4µl, alive	Single, terminal	Not reported	single	Mass spectrometry only

**Meyding-Lamade, U et al 1996[21] and 1999[22]**	mouse	“closed”, subdural	Not reported	Single, terminal	Not reported	Presumably single[22]	1x PCR for virus
**Liu L et al 2004[23]**	mouse	“closed”, subdural	7µl	Single/ repeated	Not reported	single	1x ELISA
**Liu L et al 2008[24]**	mouse	“closed”, subdural	3-15 µl	Single/ repeated	Not reported	single	2x ELISA
**Pigoni M et al 2016[25]**	mouse	“closed”, subdural	9-10µl	Single, terminal	Not reported	Single and pooled for some groups	1x WB, mass spectrometry.
**Tushaus J et al 2020[26]**	mouse	“closed”, subdural	Not reported	Single, terminal	Not reported	single	Mass spectrometry
**Lim NKH et al 2018[27]**	mouse	“closed”, subdural	10-15µl	Single, terminal	Not reported	single	1x ELISA
**Shimizu K et al 2022[28]**	mouse	“closed”, subdural	10 µl	Single, terminal	Not reported	single	Not reported
**Han et al 2023[29]**	mouse	“closed”, blind without optical verification	5-10µl	Single/ repeated	Not reported	pooled	mass spectrometry
**Kaur et al 2023[30]**	mouse	“closed”, subdural	8-15µl	Single, terminal	Hb measurement in pellet	single	Not reported
**Present study**	mouse	“closed”, subdural	19-28µl (mean 26µl)	Single/ repeated	QC establishment for mouse CSF	single	>2x WB, >2x ELISA, mass spectrometry

Supplementary table 4: DIA window scheme

Window #	Window Center (m/z)	Window Width (m/z)
1	341.5	85
2	403	40
3	437	30
4	465	28
5	491	26
6	515.5	25
7	539	24
8	562	24
9	585	24
10	608	24
11	631.5	25
12	656.5	27
13	683	28
14	710.5	29
15	741	34
16	776	38
17	817	46
18	869.5	61
19	974.5	151
20	1225	352

Supplementary table 5: Comparison of murine CSF proteomics studies. * indicates studies performed in the Lichtenthaler lab

Publication	Mouse Model	Age	CSF vol	Mass Spec	Acquisition	Software	Protein Ids
Dislich et al 2015[31]*	BACE1 KO	4M	5 µL	QE	DDA, LFQ	Maxquant	715
Eninger et al 2022[32]*	APPPS1, A30P-αS	APPPS1: 3, 18M; A30P-αS: 3, 11, 18M	5 µL	QE	DDA, LFQ	Maxquant	526-665
Pesämaa et al 2023[33]*	TREM2 KO, GRN KO	12M	5 µL	QE-HF	DDA, LFQ	Maxquant	744, 703
Tüshaus et al 2021[34]*	iRhom1 KO	3M	5 µL	QE HF	DDA, LFQ	Maxquant	669
Tüshaus et al 2020[26]*	WT C57BL/6	3M	5 µL	QE HF	DIA, LFQ	Spectronaut	984
Rother et al 2022[35]*	APPPS1 +/- BACE inhibitor	21.5M	5 µL	timsTOF pro	DIA-PASEF, LFQ	DIA-NN	2931
Gorska et al 2024[36]	APPPS1	28.6W	4 µL	timsTOF flex	DIA, LFQ	DIA-NN	1514-4479
Delvenne et al 2024[37]	APP-NL-G-F	7W, 40W	5 µL	QE HF	DIA, LFQ	Skyline	1358
Kaag Rasmussen et al 2024[38]	migraine mouse model	N/A	N/A	N/A	N/A	N/A	N/A
Smith et al 2014[20]	WT C57BL/6		4 µL	LTQ Orbitrap Velos	DDA, LFQ, 2D-LC-MS/MS	N/A	566
Sleat et al 2019[39]	lysosomal storage diseases CLN1, 2, 3	CLN1: 4, 19W; CLN2: 4, 26W; CLN3: 4, 52W	pooled CSF of 10 mice	QE HF	TMT-10plex, 20 fractions	Global Proteome Machine version 2.2.1	4012
Hsu et al 2019[40]	ischemic stroke	6W	pooled CSF of 3 mice	LTQ Orbitrap XL	DDA, LFQ	Maxquant	344
Jiang et al 2022[41]	APP NL-F, APP NL-G-F	13M	N/A	QE	DDA, LFQ	Maxquant	383
Han et al 2023[29]	WT C3H/HeJ	N/A	pooled CSF of 5 mice, 10 µL	timsTOF pro 2	DDA-PASEF, LFQ	Maxquant	513

References

1. Maetzler W*, et al.* Serum and cerebrospinal fluid levels of transthyretin in Lewy body disorders with and without dementia. *PLoS One* **7**, e48042 (2012).

2. Koch S*, et al.* Characterization of four lipoprotein classes in human cerebrospinal fluid. *J Lipid Res* **42**, 1143-1151 (2001).

3. Yamauchi K*, et al.* Apolipoprotein E in cerebrospinal fluid: relation to phenotype and plasma apolipoprotein E concentrations. *Clin Chem* **45**, 497-504 (1999).

4. Wasik N*, et al.* Clusterin, a New Cerebrospinal Fluid Biomarker in Severe Subarachnoid Hemorrhage: A Pilot Study. *World Neurosurg* **107**, 424-428 (2017).

5. Aeinehband S*, et al.* Complement component C3 and butyrylcholinesterase activity are associated with neurodegeneration and clinical disability in multiple sclerosis. *PLoS One* **10**, e0122048 (2015).

6. Winer L*, et al.* SOD1 in cerebral spinal fluid as a pharmacodynamic marker for antisense oligonucleotide therapy. *JAMA Neurol* **70**, 201-207 (2013).

7. Cuchillo-Ibanez I*, et al.* Heteromers of amyloid precursor protein in cerebrospinal fluid. *Mol Neurodegener* **10**, 2 (2015).

8. Ishii T*, et al.* Increased cerebrospinal fluid complement C5 levels in major depressive disorder and schizophrenia. *Biochem Biophys Res Commun* **497**, 683-688 (2018).

9. Garland P*, et al.* Heme-Hemopexin Scavenging Is Active in the Brain and Associates With Outcome After Subarachnoid Hemorrhage. *Stroke* **47**, 872-876 (2016).

10. Jakobsson J*, et al.* Decreased cerebrospinal fluid secretogranin II concentrations in severe forms of bipolar disorder. *J Psychiatry Neurosci* **38**, E21-26 (2013).

11. Mast H, Poche H, Marx P. Hemoglobin quantitation in subarachnoid hemorrhage. *Klin Wochenschr* **65**, 513-515 (1987).

12. Wright BL, Lai JT, Sinclair AJ. Cerebrospinal fluid and lumbar puncture: a practical review. *J Neurol* **259**, 1530-1545 (2012).

13. Tumani H*, et al.* S1 guidelines “lumbar puncture and cerebrospinal fluid analysis” (abridged and translated version). *Neurological Research and Practice* **2**, 8 (2020).

14. Barkovits K*, et al.* Blood Contamination in CSF and Its Impact on Quantitative Analysis of Alpha-Synuclein. *Cells* **9**, (2020).

15. DeMattos RB*, et al.* Plaque-associated disruption of CSF and plasma amyloid-beta (Abeta) equilibrium in a mouse model of Alzheimer's disease. *J Neurochem* **81**, 229-236 (2002).

16. Maia LF*, et al.* Changes in amyloid-beta and Tau in the cerebrospinal fluid of transgenic mice overexpressing amyloid precursor protein. *Sci Transl Med* **5**, 194re192 (2013).

17. Cunningham R, Jany P, Messing A, Li L. Protein changes in immunodepleted cerebrospinal fluid from a transgenic mouse model of Alexander disease detected using mass spectrometry. *J Proteome Res* **12**, 719-728 (2013).

18. Bacioglu M*, et al.* Neurofilament Light Chain in Blood and CSF as Marker of Disease Progression in Mouse Models and in Neurodegenerative Diseases. *Neuron* **91**, 56-66 (2016).

19. Sakic B. Cerebrospinal fluid collection in laboratory mice: Literature review and modified cisternal puncture method. *J Neurosci Methods* **311**, 402-407 (2019).

20. Smith JS, Angel TE, Chavkin C, Orton DJ, Moore RJ, Smith RD. Characterization of individual mouse cerebrospinal fluid proteomes. *Proteomics* **14**, 1102-1106 (2014).

21. Meyding-Lamade U, Ehrhart K, de Ruiz HL, Kehm R, Lamade W. A new technique: serial puncture of the cisterna magna for obtaining cerebrospinal fluid in the mouse--application in a model of herpes simplex virus encephalitis. *J Exp Anim Sci* **38**, 77-81 (1996).

22. Meyding-Lamade U*, et al.* Herpes simplex virus encephalitis: chronic progressive cerebral MRI changes despite good clinical recovery and low viral load - an experimental mouse study. *Eur J Neurol* **6**, 531-538 (1999).

23. Liu L, Herukka SK, Minkeviciene R, van Groen T, Tanila H. Longitudinal observation on CSF Abeta42 levels in young to middle-aged amyloid precursor protein/presenilin-1 doubly transgenic mice. *Neurobiol Dis* **17**, 516-523 (2004).

24. Liu L, Duff K. A technique for serial collection of cerebrospinal fluid from the cisterna magna in mouse. *J Vis Exp*, (2008).

25. Pigoni M*, et al.* Seizure protein 6 and its homolog seizure 6-like protein are physiological substrates of BACE1 in neurons. *Mol Neurodegener* **11**, 67 (2016).

26. Tushaus J*, et al.* An optimized quantitative proteomics method establishes the cell type-resolved mouse brain secretome. *EMBO J* **39**, e105693 (2020).

27. Lim NK, Moestrup V, Zhang X, Wang WA, Moller A, Huang FD. An Improved Method for Collection of Cerebrospinal Fluid from Anesthetized Mice. *J Vis Exp*, (2018).

28. Shimizu K, Gupta A, Brastianos PK, Wakimoto H. Anatomy-oriented stereotactic approach to cerebrospinal fluid collection in mice. *Brain Res* **1774**, 147706 (2022).

29. Han JR, Yang Y, Wu TW, Shi TT, Li W, Zou Y. A Minimally-Invasive Method for Serial Cerebrospinal Fluid Collection and Injection in Rodents with High Survival Rates. *Biomedicines* **11**, (2023).

30. Kaur A, Shuken S, Yang AC, Iram T. A protocol for collection and infusion of cerebrospinal fluid in mice. *STAR Protoc* **4**, 102015 (2023).

31. Dislich B*, et al.* Label-free Quantitative Proteomics of Mouse Cerebrospinal Fluid Detects beta-Site APP Cleaving Enzyme (BACE1) Protease Substrates In Vivo. *Mol Cell Proteomics* **14**, 2550-2563 (2015).

32. Eninger T*, et al.* Signatures of glial activity can be detected in the CSF proteome. *Proc Natl Acad Sci U S A* **119**, e2119804119 (2022).

33. Pesämaa I*, et al.* A microglial activity state biomarker panel differentiates FTD-granulin and Alzheimer's disease patients from controls. *Molecular Neurodegeneration* **18**, 70 (2023).

34. Tushaus J*, et al.* The pseudoprotease iRhom1 controls ectodomain shedding of membrane proteins in the nervous system. *FASEB J* **35**, e21962 (2021).

35. Rother C*, et al.* Experimental evidence for temporal uncoupling of brain Aβ deposition and neurodegenerative sequelae. *Nature Communications* **13**, 7333 (2022).

36. Górska AM, Santos-García I, Eiriz I, Brüning T, Nyman T, Pahnke J. Evaluation of cerebrospinal fluid (CSF) and interstitial fluid (ISF) mouse proteomes for the validation and description of Alzheimer's disease biomarkers. *Journal of Neuroscience Methods* **411**, 110239 (2024).

37. Delvenne A*, et al.* Involvement of the choroid plexus in Alzheimer's disease pathophysiology: findings from mouse and human proteomic studies. *Fluids and Barriers of the CNS* **21**, 58 (2024).

38. Kaag Rasmussen M*, et al.* Trigeminal ganglion neurons are directly activated by influx of CSF solutes in a migraine model. *Science* **385**, 80-86 (2024).

39. Sleat DE*, et al.* Analysis of Brain and Cerebrospinal Fluid from Mouse Models of the Three Major Forms of Neuronal Ceroid Lipofuscinosis Reveals Changes in the Lysosomal Proteome. *Mol Cell Proteomics* **18**, 2244-2261 (2019).

40. Hsu WH*, et al.* Combined proteomic and metabolomic analyses of cerebrospinal fluid from mice with ischemic stroke reveals the effects of a Buyang Huanwu decoction in neurodegenerative disease. *PLoS One* **14**, e0209184 (2019).

41. Jiang R*, et al.* Increased CSF-decorin predicts brain pathological changes driven by Alzheimer's Abeta amyloidosis. *Acta Neuropathol Commun* **10**, 96 (2022).

42. Zhang Y, et al. Purification and Characterization of Progenitor and Mature Human Astrocytes Reveals Transcriptional and Functional Differences with Mouse. Neuron 89, 37-53 (2016).

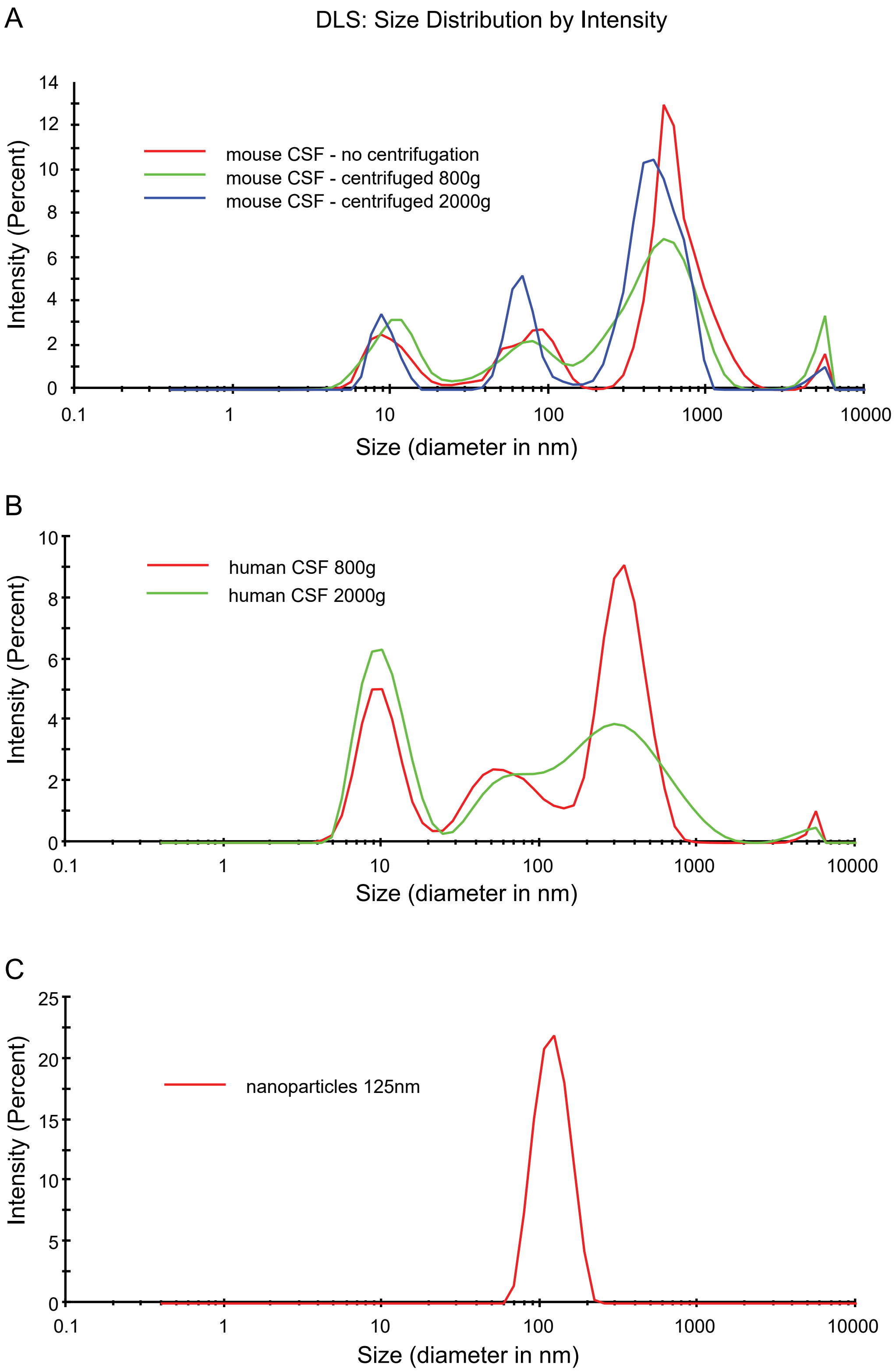

Supplementary Figure 1. Dynamic light scattering analysis of mouse and human CSF microparticles. Graphs show the size distribution of particles in mouse (A) or human CSF (B) under different centrifugation conditions. For assay quality control we use nanoparticles of a known size (C).

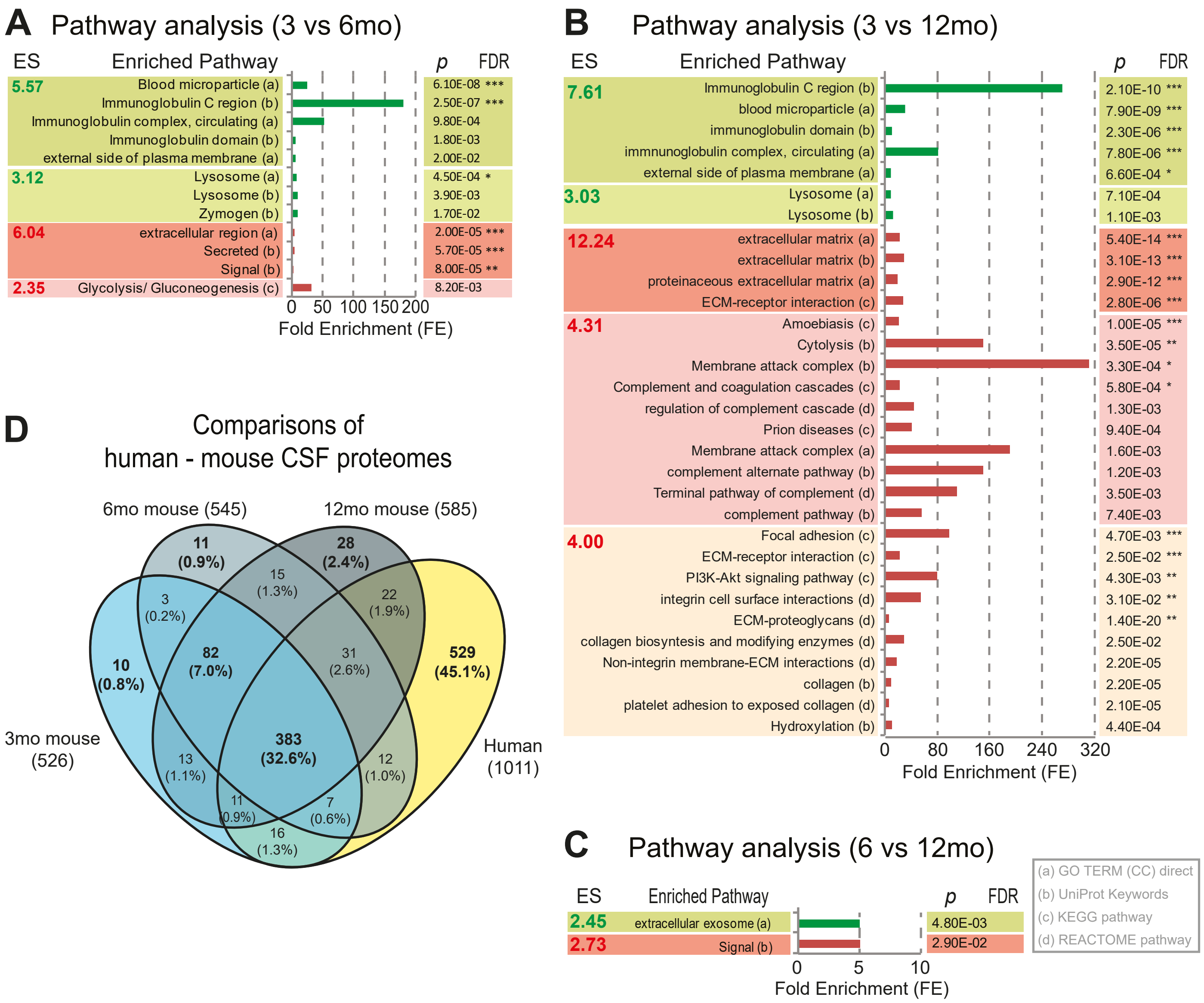

Supplementary Figure 2. Supplementary data to main Figure 2 for the analysis of single mouse CSF sampling. Pathway analysis of changed proteins between the A) 6 versus 3 months old, B) 12 versus 3 months-old and C) 12 versus 6 months-old mice, using the functional annotation clustering tool of DAVID-Database. The Enrichment Score (ES) ranks the overall importance (enrichment) of an annotation pathway in the proteome; green boxes contain the increased pathways, red boxes the decreased ones; the columns on the right indicate the corresponding p- (Benjamini correction) and FDR- (false discovery rate) significance values for each enriched (i.e. changed) pathway (*<0.05, **<0.01, ***<0.001); Fold Enrichment (FE) shows the magnitude of enrichment for each pathway (increased or decreased); the grey box next to (C) annotates via letters "a" to "d" the corresponding database for each of the enriched pathways. D) illustrative comparison (Venn diagram) of the common and different proteins between the 3 age-groups in mice and human CSF: 72.2% of the proteome remains common through aging from 3 to 12 months, the remaining 27.8% change dynamically.

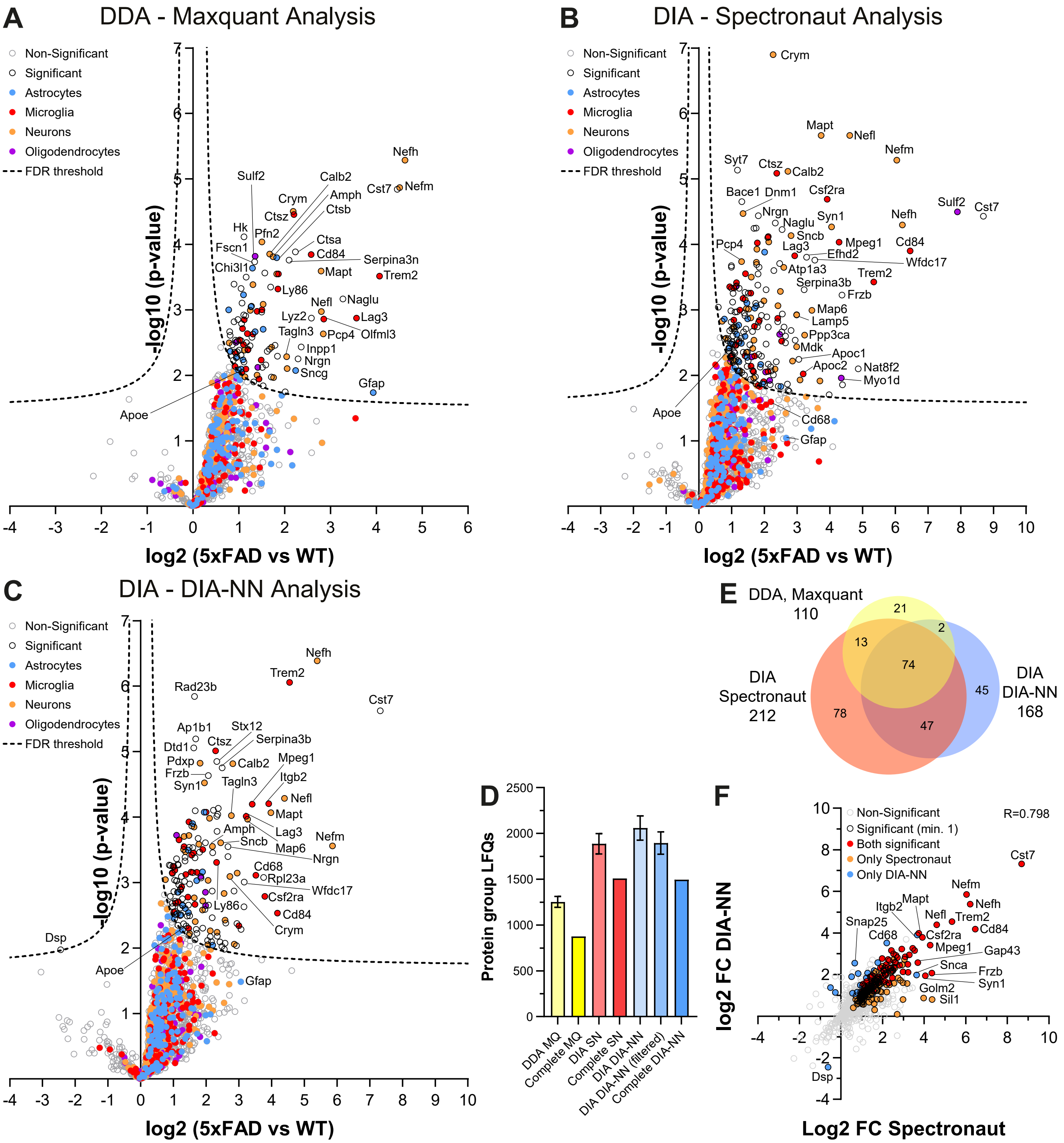

Supplementary Figure 3. A-B) Volcano plots of DDA with Maxquant (A), DIA with Spectronaut (B) and DIA with DIA-NN analysis. The minus log10 transformed p-values of each protein are plotted against the log2 protein LFQ ratios. The dashed lines indicate a permutation-based FDR correction (p=0.05; s_0=0.1). Proteins are categorized and labeled by color, based on their major cell type of origin[26, 42]. D) Protein LFQ values for DDA data analyzed with Maxquant (MQ) and DIA data analyzed with Spectronaut (SN) or DIA-NN (average +/- SD). "Complete" indicates protein groups without missing values. E) Venn digramm showing the overlap of significantly changed proteins between DDA data analyzed with Maxquant and DIA data analyzed with Spectronaut or DIA-NN. E) Detailed comparison of protein log2 fold changes between Spectronaut and DIA-NN analysis. Proteins with a significant change in at least one comparison are labled with black circles. Proteins significant in both analyses are filled with red color, wheras those only significant in the Specronaut or DIA-NN analysis are filled with orange or light blue color, respectively.

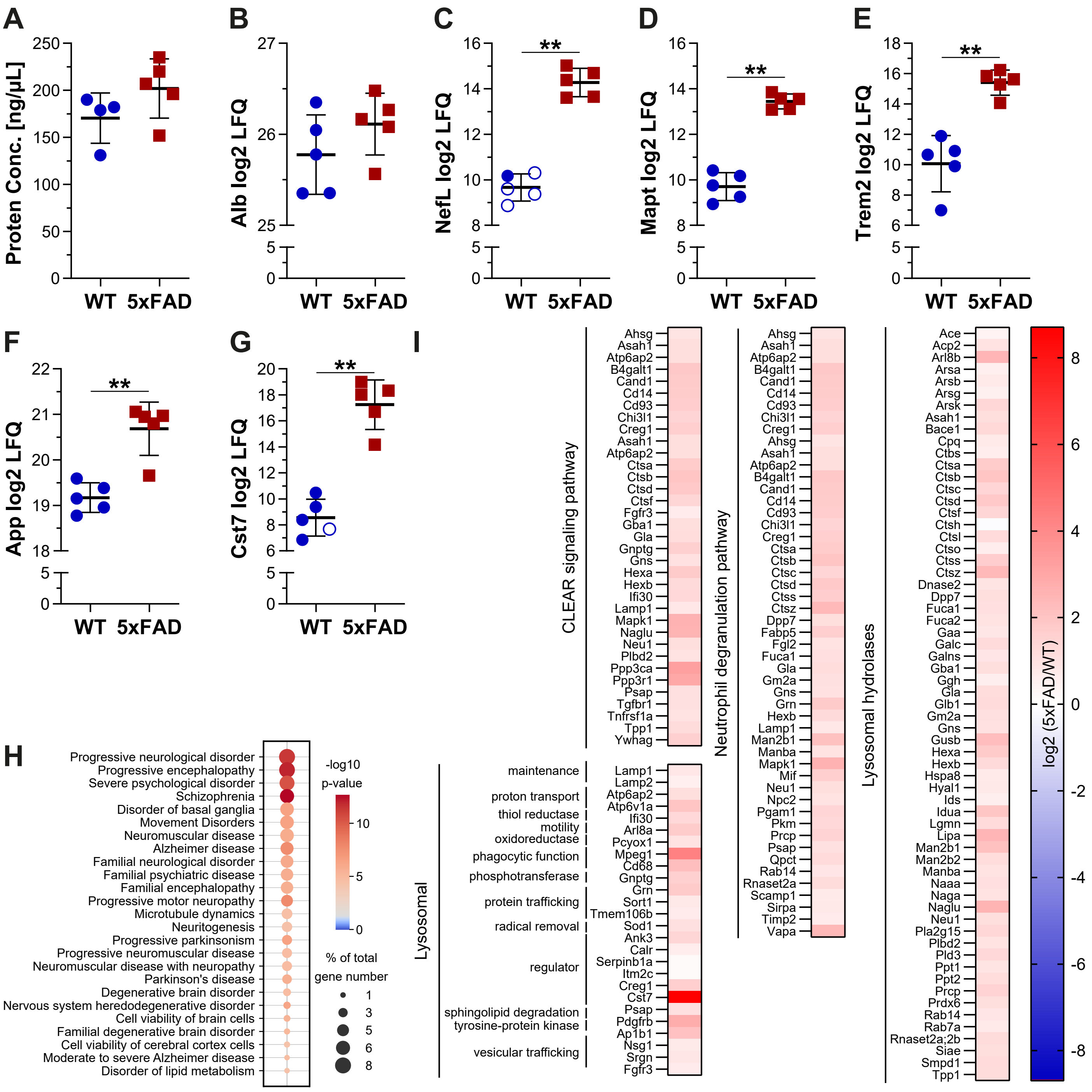

Supplementary Figure 4. A) Protein concentrations measured using the Qubit protein assay. B-G) Dot plots of log2 transformed protein LFQ intensities for selected proteins (DIA, Spectronaut analysis, WT: n=5, 5xFAD: n=5, * $p < 0.05$, ** $p < 0.01$). The average values are displayed +/- standard deviation. Empty circles indicate imputated values. H) Bubble plot of Ingenuity pathway diesease and bio functions enrichment analysis. The dot size indicates the percentage of genes in the target list matching to the different terms, whereas the color indicates the significance in -log10 scale. I) Heatmaps displaying the average log2 transformed protein LFQ ratios between 5xFAD and WT mouse CSF for Ingenuity pathways CLEAR signaling and neutrophil degranulation as well as lysosomal proteins.
